# The Effects of Health Care Chatbot Personas With Different Social Roles on the Client-Chatbot Bond and Usage Intentions: Development of a Design Codebook and Web-Based Study

**DOI:** 10.2196/32630

**Published:** 2022-04-27

**Authors:** Marcia Nißen, Dominik Rüegger, Mirjam Stieger, Christoph Flückiger, Mathias Allemand, Florian v Wangenheim, Tobias Kowatsch

**Affiliations:** 1 Centre for Digital Health Interventions Department of Management, Technology, and Economics ETH Zurich Zurich Switzerland; 2 Pathmate Technologies AG Zurich Switzerland; 3 Department of Psychology Brandeis University Waltham, MA United States; 4 Institute of Communication and Marketing Lucerne University of Applied Sciences and Arts Lucerne Switzerland; 5 Department of Psychology University of Zurich Zurich Switzerland; 6 University Research Priority Programs, Dynamics of Healthy Aging University of Zurich Zurich Switzerland; 7 Centre for Digital Health Interventions Institute of Technology Management University of St.Gallen St.Gallen Switzerland

**Keywords:** chatbot, conversational agent, social roles, interpersonal closeness, social role theory, working alliance, design, persona, digital health intervention, web-based experiment

## Abstract

**Background:**

The working alliance refers to an important relationship quality between health professionals and clients that robustly links to treatment success. Recent research shows that clients can develop an affective bond with chatbots. However, few research studies have investigated whether this perceived relationship is affected by the social roles of differing closeness a chatbot can impersonate and by allowing users to choose the social role of a chatbot.

**Objective:**

This study aimed at understanding how the social role of a chatbot can be expressed using a set of interpersonal closeness cues and examining how these social roles affect clients’ experiences and the development of an affective bond with the chatbot, depending on clients’ characteristics (ie, age and gender) and whether they can freely choose a chatbot’s social role.

**Methods:**

Informed by the social role theory and the social response theory, we developed a design codebook for chatbots with different social roles along an interpersonal closeness continuum. Based on this codebook, we manipulated a fictitious health care chatbot to impersonate one of four distinct social roles common in health care settings—institution, expert, peer, and dialogical self—and examined effects on perceived affective bond and usage intentions in a web-based lab study. The study included a total of 251 participants, whose mean age was 41.15 (SD 13.87) years; 57.0% (143/251) of the participants were female. Participants were either randomly assigned to one of the chatbot conditions (no choice: n=202, 80.5%) or could freely choose to interact with one of these chatbot personas (free choice: n=49, 19.5%). Separate multivariate analyses of variance were performed to analyze differences (1) between the chatbot personas within the no-choice group and (2) between the no-choice and the free-choice groups.

**Results:**

While the main effect of the chatbot persona on affective bond and usage intentions was insignificant (*P*=.87), we found differences based on participants’ demographic profiles: main effects for gender (*P*=.04, *η_p_*^2^=0.115) and age (*P*<.001, *η_p_*^2^=0.192) and a significant interaction effect of persona and age (*P*=.01, *η_p_*^2^=0.102). Participants younger than 40 years reported higher scores for affective bond and usage intentions for the interpersonally more distant expert and institution chatbots; participants 40 years or older reported higher outcomes for the closer peer and dialogical-self chatbots. The option to freely choose a persona significantly benefited perceptions of the peer chatbot further (eg, free-choice group affective bond: mean 5.28, SD 0.89; no-choice group affective bond: mean 4.54, SD 1.10; *P*=.003, *η_p_*^2^=0.117).

**Conclusions:**

Manipulating a chatbot’s social role is a possible avenue for health care chatbot designers to tailor clients’ chatbot experiences using user-specific demographic factors and to improve clients’ perceptions and behavioral intentions toward the chatbot. Our results also emphasize the benefits of letting clients freely choose between chatbots.

## Introduction

### Motivation

In health care, the ongoing and active engagement of clients in their treatment and care is paramount to achieving optimal health outcomes [1] (eg, in the management of chronic diseases, such as depression and diabetes) but also to promote sustained behavioral and lifestyle changes in preventive medicine. Yet, compared to other settings, such as retail or hospitality, the health care context is arguably unique in at least two respects: (1) the highly personal, sensitive, emotional, and potentially high-stakes nature of most health matters and (2) the interpersonal nature of many therapeutic approaches and clients’ encounters with various social health care roles [2].

Since personnel and financial resources of health care professionals to monitor and foster clients’ engagement in their therapy are limited [3], the development of a broad range of novel digital health technologies is increasingly supporting efforts to empower clients to take charge of their own health outside clinical settings at every stage of the patient journey. Among these digital innovations, health care chatbots (ie, text-based conversational agents), in particular, have been received with enthusiasm. Chatbots are said to simplify and “humanize” access to digital health care services, even in longitudinal settings [4,5], since they mimic natural physician-patient dialogue, where clients engage in natural, text- or voice-based interpersonal exchanges via messenger-like user interfaces [6]. Taking over the role of “digital coaches” [7], they can develop an *affective bond* with clients and support them in their everyday lives and therapeutic settings beyond on-site consultations anywhere and anytime [8]. However, maintaining engagement with a chatbot aimed at fostering patient engagement brings new challenges and raises the question of which design choices foster engagement and an affective bond with the chatbot in the first place [9,10].

Originally developed for psychotherapeutic settings, the working alliance—often also referred to as the therapeutic or helping alliance—is a key construct in the therapist-client collaboration in the clinical context of mental health disorders, reflecting the collaborative quality between clients and health professionals [11]. The working alliance is robustly linked to patient engagement, retention [12,13], and therapeutic success [14,15] and encompasses three subdimensions [16]: *therapeutic goals* and *therapeutic tasks* that the health professional and client jointly agree upon “in the context of an *affective bond* or positive attachment” [17]. This affective bond subsumes a sense of sympathy, interpersonal closeness, familiarity, trust, or common purpose and understanding between a mental health professional and a client [14]. In social psychology, bond, relatedness, attachment, intimacy, and closeness between two social actors are often subsumed under the psychological concept of interpersonal closeness [18]. The development of interpersonal closeness and a sustainable affective bond between mental health professionals and clients strongly depends on continuous personal encounters in either face-to-face psychotherapy [14,15], online therapy [19,20], or remote consultations [21].

A recent study among 36,070 users of the text-based chatbot Woebot found that clients may develop a working alliance with a chatbot within 5 days [22]. However, while a body of work describes how or which design choices affect the development of an affective bond with *embodied* conversational agents (which can use more nonverbal cues such as mimicry and gestures to convey interpersonal closeness [23]), it is still unclear which design choices foster an affective bond with *text-based* chatbots [24].

Research under the social response theory—also known as the *computers are social actors* paradigm or the media equation—has demonstrated that individuals anthropomorphize conversational agents that exhibit human-like social cues [25], treat them as social actors [26], and are capable of developing relationships with them [22]. Previous work suggests that a chatbot’s social role constitutes one such cue that could “cause people to make inferences about social presence in a computing product” [27], which could help elicit social responses [28].

However, in practice, chatbots are often mindlessly designed “to perform social roles traditionally associated with humans” [29], for instance, a therapist role, in the case of Woebot, or a nurse role, in the case of the Florence chatbot. Theoretically, however, it is not yet well understood how a chatbot’s impersonated social role actually affects a client’s relationship with the chatbot and, thus, the affective bond between the client and the chatbot and the client’s intentions to use the chatbot.

With this study, we aimed to close this gap by, first, investigating which design choices allow the manifestation of the social role of a chatbot (research question 1) and how a chatbot’s social role affects users’ affective bond with the chatbot and their intentions to use it (research question 2). Second, we explored how an individual’s demographic profile (ie, gender and age; research question 3) and the option to freely choose the social role of a chatbot affect these evaluations (research question 4).

### Designing Engaging Health Care Chatbots With Human-Like Social Roles

Social role theory “concerns itself with a triad of concepts: patterned and characteristic social behaviors, parts or identities that are assumed by social participants, and scripts or expectations for behavior that are understood by all and adhered to by the performer” [30]. Social role theory has long been applied in health care to disentangle communication and power dynamics in the physician-client relationship [31,32] and to better understand how specific social communication scripts are used by physicians and clients to navigate their different social roles and interactions [33-37].

Considering chatbots as social actors (cf, social response theory), individuals can be expected to apply readily available learned human social scripts to interactions with a chatbot as well, especially when specific cues signal that it enacts a particular role [27,38]. Prior conceptual conversational agent studies have developed taxonomies, typologies, and classifications of different types of chatbots, for example, differentiating chatbots for domain-specific or for general-purpose use [39], for specific applications (eg, business-to-business customer services [40] and health care [41]), for different purposes [42,43], for single- or multiple-user use cases [44,45], or for specific periods [46]. However, relatively few conceptual studies to date have addressed how a chatbot can impersonate a holistic, domain-specific social role and how such a social role affects user assessments. In e-commerce contexts, McGoldrick et al [47] identified and investigated three possible roles for virtual sales agents (ie, friend, personal buyer, or helper) and found that the helper role was most widely preferred, followed by the friend role. Similarly, Rhee and Choi [48] investigated effects of two possible roles (ie, friend or secretary) for a voice-enabled recommendation agent and found that consumers rated a product more favorably when it was recommended by a friend role. Another study on perceptions of Apple’s voice assistant Siri found that participants expressed more favorable attitudes toward Siri when they thought of it as a coworker versus a supervisor or a friend [49]. In digital health care settings, however, we are not aware of any previous study that has investigated how individuals’ attitudes toward social roles that are impersonated by a health care chatbot promote or antagonize the development of an affective bond with the chatbot and their intention to use it.

The American Psychological Association Dictionary of Psychology defines a social role as “the set of attitudes and characteristic behaviors expected of an individual who occupies a specific position or performs a particular function in a social context, such as being a spouse or acting as a caregiver for an aging parent” [50]. To this end, social role theory suggests that the perception of a particular social role is triggered by a set of role-typical social cues (eg, use of jargon and business attire) that individuals subconsciously look for to orient themselves and to understand potential outcomes or goals of a relationship with one another [51]. To fulfill a social role in a particular encounter—be it a client with a therapist, a nurse with a client, a boss with employees, etc—people mindlessly exhibit ritualized social behaviors that they have learned to be appropriate for that type of encounter [51].

Typical social roles that clients encounter in health care settings in the real world include, for example, the medical experts of their condition, supportive peers with the same condition, the institution providing the context for their health care services, and the clients themselves who have to adopt their own social roles to this situation. The conceptualization and operationalization of the latter role draws from people’s tendency to talk to themselves through internal dialogues (ie, to their “dialogical selves”). This is because intrapersonal communication is known to provide self-regulatory functions through monological, goal-directed self-talk (eg, “I believe in you!”) or more contemplative and reflective internal dialogues with one or more imaginary interlocutors [52]. Overall, these social roles can be conceptualized on an interpersonal closeness continuum, ranging from extremely distant (ie, the institution), rather distant (ie, expert), and rather close (ie, peer) to extremely close (ie, the client’s dialogical self) social roles.

Social, interpersonal closeness cues that are available to conversational agents include (1) visual cues (eg, the avatar), (2) verbal style cues (eg, form of address), (3) nonverbal style cues (eg, emojis), and (4) verbal relational content cues (eg, self-disclosures and jokes) [53]. To provide an overview of empirical studies of interpersonal closeness cues and their effects, we conducted a systematic analysis of the literature to date. Specifically, 116 research articles that were included in two recently published systematic literature reviews on design features of embodied conversational agents [23] and of text-based chatbots [24] were analyzed with regard to the types of cues and the outcome variables investigated in the included studies, respectively (Tables S1 and S2 in Multimedia Appendix 1). Articles were included for detailed analysis if they presented at least one empirical or experimental study that investigated the effects of interpersonal closeness cues on user assessments or the client-agent relationship. Studies that, for example, compared a chatbot with a website or assessed the effects of overall system quality on users’ evaluation of conversational agents were excluded from the analysis. In total, 47 studies could be analyzed: 32 studies on embodied conversational agents and 15 studies on text-based conversational agents. Tables S1 and S2 in Multimedia Appendix 1 yield descriptive details of all included studies and the classification result.

Quantitative analysis has demonstrated that the majority of all studies (76.6%) either investigated the effects of visual cues, such as abstract versus human avatars [54]; role-appropriate attire [55,56]; gender, age, or ethnicity of the embodied character (eg, Alsharbi and Richards [57]); or nonverbal style cues, such as empathic versus nonempathic facial expressions [58,59] or emoji (eg, Beattie et al [60] and Fadhil et al [61]). Another considerable share representing 46.8% of all studies analyzed the effects of verbal relational content cues, such as self-disclosures (eg, Ho and Hancock [62] and Kang and Gratch [63]) or empathic feedback (eg, Liu and Sundar [64]). Similarly, 44.7% of the articles investigated verbal style cues, such as the form of address (eg, Bickmore and Picard [65]), T/V (*tu/vos*) distinction (ie, formal and informal forms of the second-person pronoun “you” [66]), or paralinguistic and backchanneling cues [67].

While some of these articles investigated combinations or interactions of such cues, no previous study has investigated how they can be used to design holistic chatbot personas that are modeled to impersonate a particular human social role based on the adaption of various interpersonal closeness cues.

By unifying social role theory and social response theory at this point, *hypothesis 1* is as follows: It is expected that health care chatbots can be designed such that they impersonate social roles of differing closeness by using combinations of purposefully selected and differently manifested interpersonal closeness cues.

Given that the interpersonal closeness construct is closely related to the affective bond subgoal of the working alliance, and given that interpersonal closeness can be expected to be at least partially determined by the social role that a chatbot adopts in the encounter with a client, *hypothesis 2* is as follows: It is expected that chatbots that impersonate a closer social role (ie, peer or dialogical self), as compared to a more distant social role (ie, expert or institution), will improve perceived interpersonal closeness, the affective bond with the chatbot, and intentions to use the chatbot.

### Personalization and Customization of Health Care Chatbots

While personal characteristics of an individual, such as demographic factors [68] or their innate tendency to anthropomorphize objects [69], may decisively influence their capacity to experience interpersonal closeness, in chatbot design, all too often “one-fits-all approaches” still prevail [70]. Yet, personalization and customization are common strategies that companies worldwide are adopting to account for personal characteristics or preferences [71,72].

Personalization refers to the automatic tailoring of service offerings, for instance, to preferences, past usage behaviors [73], or demographic characteristics of customers [70,74], “usually based on previously collected customer data” [72]. While the personalization of chatbots to user characteristics is still in its infancy, it has already been linked to greater user satisfaction, user engagement, and dialogue quality [75] and has been deemed as important for adapting conversational agents to the changing needs of patients through the various stages of a disease [76].

Given that demographic characteristics (ie, age and gender) have been found to affect user assessments of conversational agents in inconsistent ways [47,68,74], *hypothesis 3* is as follows: It is expected that the demographic profile (ie, gender and age) will affect perceived interpersonal closeness, the affective bond with the chatbot, and intentions to use the chatbot.

Customization occurs when individuals have the opportunity to proactively choose between different options, such as different chatbot roles or specific design elements within them [72]. It is generally known for other technologies that the imposed use of self-service technologies (eg, imposed information retrieval via ticketing machines or the internet) has a strong negative impact on users’ evaluations and intentions to adopt such a technology [77] and that customization options are linked to greater customer satisfaction and loyalty [78].

Although the link between free choice and perceptions or relationship-building processes with conversational agents has not previously been established, *hypothesis 4* is as follows: It is expected that free, versus imposed, choice will increase, versus decrease, users’ perceived interpersonal closeness, the affective bond with the chatbot, and intentions to use the chatbot.

### A Design Codebook for Chatbots With Different Social Roles

#### Overview

To answer our first research question and as a prerequisite to empirically assess the effects of chatbots’ impersonated social roles on perceived interpersonal closeness, the affective bond, and the intention to use them, we reviewed literature from social psychology, communication, and human-computer interaction research to develop a design codebook for chatbots with different social roles. A prior version of the design codebook and the study design have been presented at the European Conference on Information Systems 2018 (ECIS 2018) and published as research-in-progress work in the conference proceedings [79].

Given our focus on chatbots that operate in health care settings and given that the affective bond plays a central role in the physician-client relationship [80], we were eager to develop design guidelines that would allow us to manipulate interpersonal closeness with chatbots. Overall, we modeled four distinct chatbot personas embodying typical social roles that clients encounter in health care settings on the proposed interpersonal closeness continuum; the personas were as follows: an institution‑like chatbot, an expert‑like chatbot, a peer‑like chatbot, and a dialogical self–like chatbot. We define chatbot personas as “composite archetypes” of their corresponding human social roles impersonated by the chatbots by different combinations and manifestations of selected interpersonal closeness cues [81].

**Figure 1:**
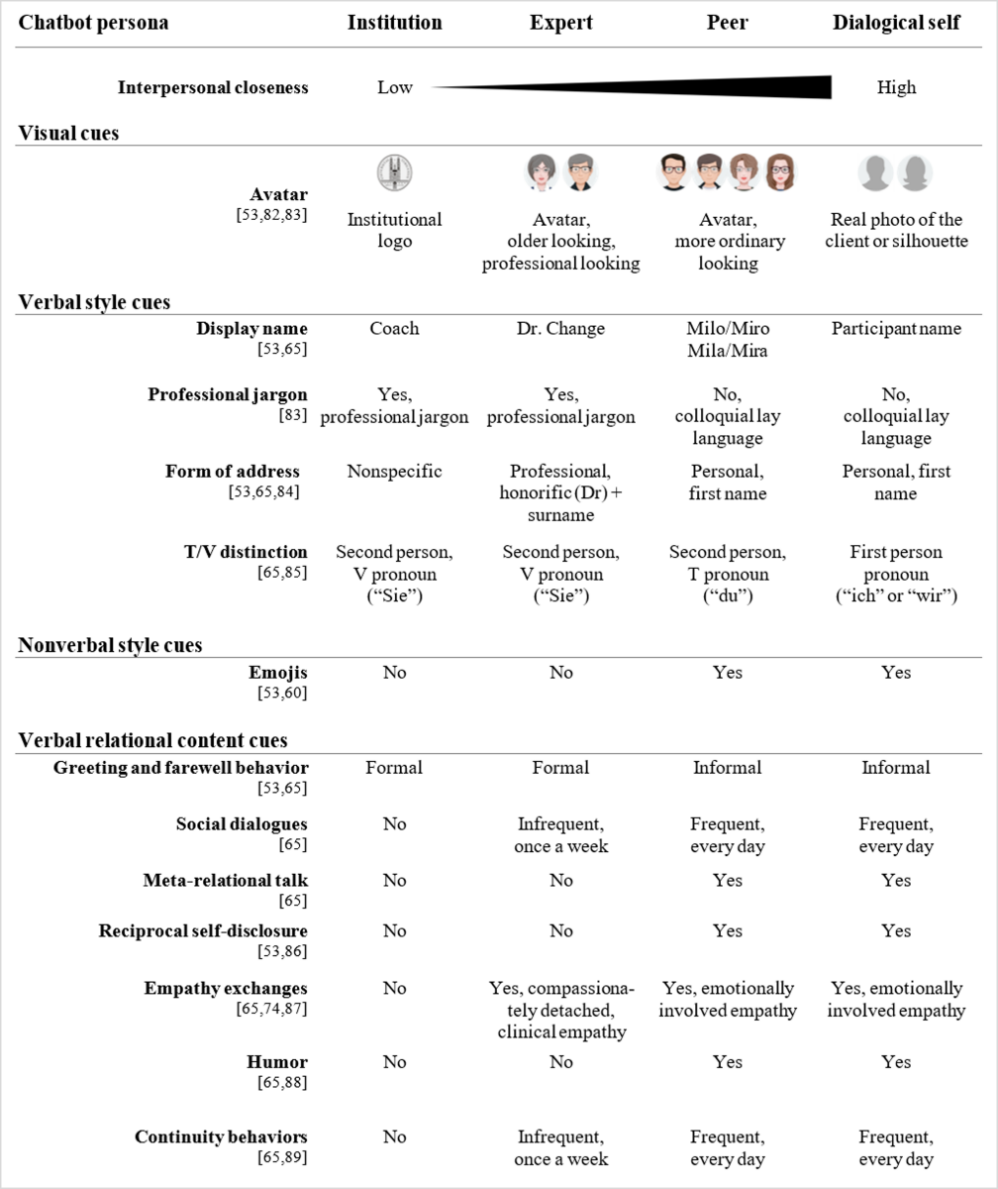
Design codebook for chatbots with different social roles. A research-in-progress version of this design codebook had been presented at the European Conference on Information Systems 2018 and published in the conference proceedings. The version in this paper represents the latest version. The T/V distinction design cue is only applicable to chatbots that operate in a language in which different pronouns are used for different social contexts; stated pronouns here are in German. T/V: *tu/vos*.

As a base for the development of a design codebook for chatbots with different social roles, we included seven out of eight suggested “behaviors” from the framework of relational behaviors for embodied conversational agents by Bickmore and Picard [65]: form of address, politeness, social dialogue, meta-relational dialogue, empathy exchanges, humor, and continuity behaviors. We did not include nonverbal immediacy behaviors, which are only applicable to embodied conversational agents. We then compared Bickmore and Picard’s [65] framework against the taxonomy of social cues by Feine et al [53] to organize all interpersonal closeness cues into four groups (ie, visual cues, verbal style cues, nonverbal style cues, and verbal relational content cues) and included six new cues from their taxonomy (ie, avatar, display name, professional jargon, emojis, greeting and farewell behavior, and self-disclosures). In total, 13 interpersonal closeness cues were included in the design codebook for chatbots with different social roles (Figure 1 [53,60,65,74,82-89]).

To develop the chatbot personas, we followed Bickmore and Picard’s [65] approach and defined how these cues would manifest for each chatbot social role, having in mind the social role theory, social response theory, and the interpersonal closeness continuum along which we had allocated the four social roles. In the following sections, we will outline, per cue category, why we included a particular interpersonal closeness cue and how we derived the different manifestations of each interpersonal closeness cue per chatbot social role.

#### Visual Cues

Since our research focuses on chatbots that operate in a text-messaging format, we only considered one visual cue: a static avatar. Manipulating the graphical representation of the chatbot persona allowed us to leverage findings from prior research. This research found priming effects of visual cues triggering social and contextual psychological and behavioral reactions [83], which can reduce uncertainty about the chatbot’s role and one’s own role in an initial interaction, similar to human-human initial encounters, where individuals rely on visual cues to form first impressions of one another [82]. Accordingly, we designed the avatars to convey stereotypical features of their roles by altering age and accessories (eg, glasses) of the avatars to make them look more professional (ie, expert) or ordinary (ie, peer). For the institution-like chatbot, a logo of the university, where the chatbot prototype had been developed, was used. In the dialogical-self condition, participants had the chance to either upload a photo of themselves or to use a default representation of a gendered human-resembling silhouette.

#### Verbal Style Cues

Verbal style cues are a key aspect for conveying important social and contextual information and, therefore, for facilitating social understanding [90] and, thus, for the creation of artificial agents [53]. Accordingly, we included three verbal style cues available to text-based chatbots: form of address, professional jargon, and T/V distinction [66].

Form of address is considered a sociolinguistic cue that verbally conveys the degree of formality and politeness imposed by the relational closeness between two interlocutors, a phenomenon known as “social deixis” and widely studied in pragmatics and sociolinguistics research [85]. Accordingly, we chose a professional formal address for the expert (ie, honorific [eg, Prof or Dr] + surname; here, “Dr. Change”) compared to the more personal, informal form of address by the peer version and the dialogical-self version, who would address users by their first name only (eg, “Hey David”). The institution version was designed to completely avoid the use of direct address and used indirect and passive constructions (eg, “Please indicate how you feel today”).

Similar to the form of address, T/V distinction is considered as another socially deictic cue [85]. This cue refers to the use of distinct second-person pronouns to denote less (ie, T form, Latin: “*tu*”) or more (ie, V form, Latin: “*vos*”) formality, power, or intimacy, depending on the relational status between two interlocutors [66]. Accordingly, since the study was conducted among a German-speaking population, the institution and expert chatbot used the German V form “*Sie*,” whereas the peer and dialogical-self chatbot used the German T form “*du*.”

Professional jargon refers to the use of a learned and shared, specialized language used within specific occupational groups, which can carry social and relational information about its generator [83]. Accordingly, we implemented the institution- and expert-like chatbots using more abstract, medical language (eg, translated from German: “Please read the following instructions for today’s exercise carefully” and “Thank you very much, I will put the note in your files”), whereas the peer- and dialogical self–like chatbots used more colloquial and lay language (eg, “Then I’ll explain today’s task to you right away” and “Thank you, got it!”).

#### Nonverbal Style Cues

Generally, computer-mediated communication lacks the possibility to convey classical nonverbal style cues, such as gestures or gaze. However, since nonverbal cues are “vital to interpersonal processes [to convey and interpret] feelings and attitudes” [60], we included emojis as available nonverbal cues available in computer-mediated communication. Prior research has shown that the use of emojis (ie, icons displaying emotions, also known as “smileys”) has the potential to surrogate other nonverbal cues [60] and that they are indicative of the development of close interpersonal relationships [91]. Accordingly, the peer and dialogical-self chatbots will make use of emojis, whereas the institution and expert chatbots will not.

#### Verbal Relational Content Cues

Five verbal relational content cues were directly adopted from Bickmore and Picard’s [65] framework of relational behavior: social dialogue, meta-relational talk, empathy exchanges, humor, and continuity behaviors. Additionally, we included greeting and farewell behavior and reciprocal self-disclosure as a potential strategy to increase interpersonal closeness, which Bickmore and Picard [65] had discussed but not integrated into their framework. Yet, Feine et al [53] included them in their taxonomy as well.

Greeting and farewell behavior directly reflects the degree of formality and politeness imposed by the social relationship between two interlocutors (see Laver [84] as cited in Bickmore and Picard [65]) and, accordingly, was manipulated gradually from very formal (ie, institution) to very informal (ie, dialogical self).

Social dialogue refers to the use of small talk, which, “on the surface, [may] not seem to move the dialogue forward at all” but is essential “to how humans obtain information about one another’s goals and plans and decide whether collaborative work is worth engaging in at all” [92]. Since the depth and breadth of social dialogue are indicative of the level of trust and familiarity between two interlocutors [92], the institution-like chatbot will not engage in any social dialogue, the expert-like chatbot will engage in it only once, and the peer- and dialogical-self chatbots will use it frequently to transition between more task-oriented talk and goal-oriented talk.

Meta-relational talk entails communication about the relationship, such as “discussing the nature of the relationship [or] disclosing one’s desires for the relationship” [93]. Research comparing the use of meta-relational talk between friends, lovers, relatives, and others found that the more intimate the relationship, the more individuals talk *about* their relationship [94]. We, thus, included meta-relational dialogues only in the peer and dialogical-self chatbots.

Reciprocal self-disclosure refers to the process of reciprocally revealing increasingly personal and intimate information about oneself (eg, personal experiences, beliefs, and values). Social penetration theory posits that relationships between humans progress based on how much (ie, breadth) and how intimately (ie, depth) two interlocutors reciprocally disclose information to each other [95]. Hence, self-disclosing was found to be closely linked to liking in human-human relationships [96] as well as in human-chatbot interactions [26]. Accordingly, we included self-disclosures by the chatbots and opportunities for the users to disclose something about themselves in the peer and dialogical-self chatbots but not in the institution and expert chatbots.

Empathy exchanges refer to conveying a sense of understanding and warmth and have been described as “one of the core processes in building and maintaining relationships” [65]. However, while the clinical empathy afforded by physicians is, at best, of a professional nature characterized by “compassionate detachment...keeping sympathy at a reasonable distance to maintain an emotional balance” [97], empathy exchanges in intimate relationships are characterized by more emotional involvement and labor [98]. Accordingly, we model the expert chatbot to display a certain degree of clinical empathy and the peer and dialogical-self chatbots to express empathic concerns in an emotionally more involved fashion.

Humor refers to the use of “incongruous [comments] that [are] recognized by the receiver as an attempt to amuse and that succeeds at amusing” [88]. Since humor and laughing are known to shape interpersonal bonds and liking [99], we manipulated the peer and dialogical-self chatbots to make some self-directed, innocent jokes.

Lastly, continuity behaviors refer to actions aimed at establishing and “[maintaining] a sense of persistence in a relationship” [65], for example, by making retrospective references to the past (eg, “Last time we...”) or prospective statements about future encounters (eg, “Next time we...”). Since continuity behaviors are closely linked to relational satisfaction [89] and differ in type and frequency between strangers and friends [100], the peer and dialogical-self chatbots will always (ie, on the simulated day of the intervention in the study) use adequate continuity statements, and the expert chatbot will do so occasionally with less emphasis.

## Methods

### Study Design

We conducted a web-based experiment to investigate the effects of a health care chatbot’s social role (ie, hypothesis 2), the role of participants’ demographic profiles (ie, gender and age; hypothesis 3), and free choice of a chatbot persona (ie, hypothesis 4) on perceived interpersonal closeness, the development of an affective bond, and intentions to use a chatbot. Following the Checklist for Reporting of Results of Internet E-Surveys [101], we will outline the study design and procedure in detail.

**Figure 2:**
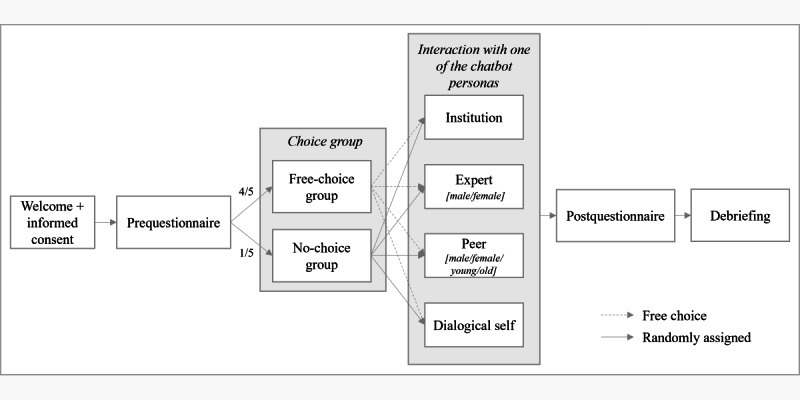
Study design. Participant numbers are listed on the arrows going into the choice groups.

The experimental design corresponded to a 4 (chatbot personas: institution, expert, peer, or dialogical self) × 2 (participant gender: female or male) × 2 (participant age: younger or older) between-group design. The sample was further divided into two experimental subgroups: (1) 4 out of 5 (80%) participants were randomly assigned to the “no-choice group,” in which they were randomly assigned to one of the chatbot conditions, introduced to the scenario (Figure S1 in Multimedia Appendix 1), and then presented with the role description (Table 1), and (2) 1 out of 5 (20%) participants were assigned to a “free-choice group,” in which they could read the descriptions first and then freely choose a chatbot persona themselves thereafter (Figure S2 in Multimedia Appendix 1). Both groups were then asked to interact with their assigned or self-chosen chatbot. Figure 2 illustrates the study design.

### Procedure

Participants were recruited via the online panel provider Talk Online Panel and rewarded based on the provider’s points-based incentive system to compensate for their efforts. The entire study was conducted in Germany in July 2017. Participants were sent an anonymous link to the closed survey via email by the panel provider. After providing informed consent regarding the study conditions, participants were screened for eligibility (ie, 18 years of age or older and native German speakers).

After completing an introductory questionnaire asking for demographic and socioeconomic data, participants were introduced to a scenario (Figure S1 in Multimedia Appendix 1) in which they were asked to test a health care chatbot prototype that promoted a personality change intervention. A digital lifestyle intervention allowed us to work with a more heterogeneous sample of healthy individuals who did not have to imagine themselves being chronically ill.

After the interaction with the respective chatbot, participants were redirected to complete the postquestionnaire, with all outcome variables and manipulation checks, and debriefed as to the experiment’s purpose. On average, participants spent 15 to 62 minutes completing the survey (mean 30.23, SD 9.13 minutes).

### Development of the Experimental Stimuli

The experimental stimuli were designed based on a prototype of a fictitious health care chatbot promoting a personality change intervention adapted from Stieger et al [102]. For purposes of standardization, the interaction with the chatbot was purely text based (ie, no voice input or output) and followed a rule-based conversational script with predefined answer options. The content of the conversation was based on fictitious scripts for the “first 2 days” and the “last day” of the intervention, which allowed us to encompass essential elements of a comprehensive health intervention (eg, introduction, goal agreement, task agreement, and feedback). All coaching elements were designed in an expert coaching style, which refers to a type of instructional coaching by an experienced coach who helps a “coachee” through providing her or him “with self-assessment tools and constructive feedback” [103]. A bidirectional peer coaching–style intervention would require similar levels of experience and mutual, reciprocal learning goals [103], which does not apply to our scenario.

We first created a minimum viable “skeleton” version of the conversational script encompassing exemplary interventional elements. In a second step, to induce the chatbots’ respective social roles, we systematically manipulated the script with respect to the manifestations and frequency of the interpersonal closeness cues defined in the conceptually derived codebook (Figure 1). Except for the different avatars, the experimental manipulation only concerned adaptations of the conversational script by adding, editing, or deleting statements and specific terms or emojis.

In the survey, all personas were introduced as follows (translated into English): “[chatbotName] is a ‘digital coach’ developed by researchers, experts, and psychotherapists from the University of Zurich, which has been equipped with various skills based on the latest findings from many years of research on ‘personality development.’” This sentence was followed by individual role-specific descriptions of the personas, as visible in Table 1.

**Figure 3:**
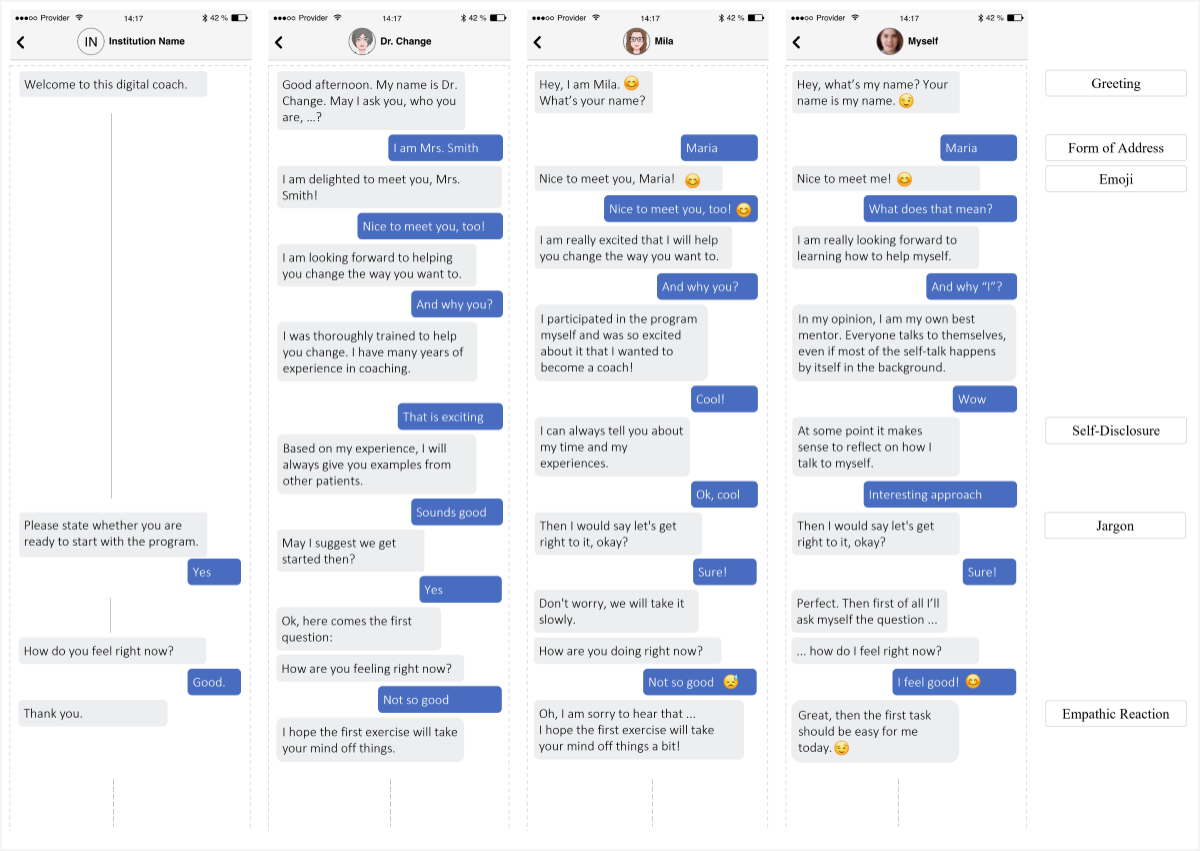
Exemplary excerpts of participants’ onboarding conversations with the four chatbot personas. The content in this figure has been translated into English by the authors for this paper.

With most interpersonal closeness cues being absent in the institution condition, the script in this condition was significantly shorter than in all other conditions (institution: 51 chatbot statements and 32 user responses; expert: 73 chatbot statements and 46 user responses; peer: 77 chatbot statements and 48 user responses; and dialogical self: 74 chatbot statements and 45 user responses; *χ*²_3_=10.5, *P*=.02). Still, an analysis of variance (ANOVA) revealed that there were no significant differences (*P*=.35) between treatment groups with regard to the average interaction time spent with the respective chatbot (mean 16.0, SD 7.11 minutes). Since interaction with the chatbot was self-paced and unsupervised, this speaks for participants’ comparable involvement with all chatbots across conditions. Figure 3 depicts excerpts of the onboarding conversations with the four chatbot personas.

**Table 1:** Chatbot personas and their introductions within the conversations.

Chatbot persona	Introduction^a^
Institution	“The PersonalityCoach has been programmed to represent the Psychological Institute of the University of Zurich, which enjoys a very good reputation worldwide in the field of personality research.”
Expert	“Dr. Change has been programmed to represent a professional psychotherapist who has many years of experience in the field of personality coaching.”
Peer	“Milo/Mila has been programmed to represent a peer who had once been a participant in the PersonalityChange program him/herself and who will share his/her own experiences with you.”
Dialogical self	“Your MySelfCoach has been programmed to give you the impression of talking to yourself through simulated inner dialogues and, thus, to help you see your experiences in a new light.”

^a^Translated into English by the authors for this paper.

### Technical Implementation

The whole study was implemented with the web-based survey tool SoSci Survey. For full customization of the health care chatbots, we built our own prototype chat app with the open-source software platform MobileCoach [104]. The chat app was then deployed in a web-based smartphone simulator using the virtualization service Appetize.io. Hence, the app could be displayed within the questionnaire but on a dedicated, separate survey page; participants did not have to download and install an app on their phone but could focus on the web-based interaction and seamlessly continue within the survey. For a screenshot of the study environment and the study prototype see Figure S3 in Multimedia Appendix 1.

Usability tests were conducted by one computer engineer, two chatbot researchers, and two domain experts who qualitatively assessed and confirmed the realism of the chatbot personas and the study design. Moreover, the complete study setup was pretested with 22 previously uninvolved individuals from the authors’ networks over two iterations to ensure comprehensibility and technical functionality.

### Sample Characteristics

The power analysis was informed by the few existing studies in the field on embodied conversational agents that had investigated the effects of relational cues on affective bond and intention to use [65]. A priori power analysis using G*Power (version 3.1; Heinrich Heine University Düsseldorf) suggested that we would need to recruit 250 participants to find medium-sized effects (*f*=0.25) in ANOVAs of between-group fixed effects at the α=.05 level of significance and statistical power of 0.80. After data runs, 251 responses were included in the final analyses (free-choice group: n=49, 19.5%; no-choice group: n=202, 80.5%). Participants ranged in age from 19 to 65 years (mean 41.15, SD 13.87 years), and 57.0% (143/251) of the total sample were female.

### Measurements

All measurements were adapted from established multi- or single-item scales; perceived interpersonal closeness was measured using the Inclusion of Other in the Self scale [18], an established and reliable pictorial instrument to measure the subjectively perceived closeness of a relationship [105]. Affective bond (Cronbach α=.900) was measured based on the bond subscale of the Working Alliance Inventory for technology-based health care interventions [106]. Intention to use (α=.952) was measured based on a scale adapted from the technology acceptance model [107]. The full list of measurements is provided in Table S3 in Multimedia Appendix 1. Manipulation check items were measured at the design cue level and carefully drafted and pretested to capture perceptions of each manipulated interpersonal closeness cue individually (Table S4 in Multimedia Appendix 1). Demographics encompassed participants’ age, gender, and native language.

### Statistical Analysis

Where constructs consisted of multiple items, reliability analyses were carried out to discern Cronbach α with all constructs scoring greater than the .70 threshold [108]. To test the main and interaction effects of the chatbot persona, participant gender, and participant age on all outcome variables (ie, hypotheses 2 and 3), one multivariate ANOVA (MANOVA) with type III sum of squares was conducted, with partial eta squared (*η_p_*^2^) indicating the size of the effect. A second MANOVA was conducted to test the effects of choice type, participant gender, and participant age on all outcome measurements (ie, hypothesis 4). Participant age was dummy coded using median split: 1=younger than 40 years (n=122, 48.6%) and 2=40 years or older (n=129, 51.4%). Where significant effects were discerned, univariate ANOVAs per outcome measurement were conducted, followed by Bonferroni-adjusted pairwise comparisons following the procedure described by Field et al [109]. Due to the mixed levels of our hypotheses on main and interaction effects, where significant interactions occurred, we investigated the highest-order interactions and not the lower-order interactions or main effects [110], also following guidance outlined in Field et al [109].

### Ethical Considerations

To meet ethical standards, we applied the following procedures. Before the study started, all participants received written information about the research project, benefits, and risks of participation. Furthermore, they were informed about their right to withhold or revoke their consent without giving reasons, their right to withdraw from participating in the study at any time during the study, and their right to receive information at any time in response to further questions when contacting the study team. They also received transparent communication about the main sources of financing for the research project as well as transparent communication that the chatbot they would be interacting with during the study was only a minimal viable prototype. Informed consent was obtained before assessment and before interaction with the prototype. At the end of the study, participants were debriefed regarding the study’s actual purpose.

According to the ETH Zurich’s Ethics Commission’s Compliance Guide regarding human subject research [111], this study did not require ethics approval for the following reasons:

- Besides participants’ age and gender, we did not collect personal information. Other social and behavioral data collected in our study were collected completely anonymously. Age and gender information are always only reported in aggregated, anonymous ways.
- Vulnerable or dependent groups were explicitly not included.
- Experimental manipulation on the chatbot did not affect functional aspects of the intervention, but only affected style- and design-related features of the chatbot.
- Experimental manipulations being researched were not likely to upset or disturb participants and did not use socially sensitive topics as a basis for the scenario development.

## Results

### Manipulation Check

Separate ANOVAs confirmed significant differences between the chatbot personas for the form of address (*P*=.02, *η_p_*^2^=0.052), professional jargon (*P*=.006, *η_p_*^2^=0.063), T/V distinction (*P*<.001, *η_p_*^2^=0.578), small talk (*P*=.003, *η_p_*^2^=0.070), self-disclosure (*P*<.001, *η_p_*^2^=0.289), use of emojis (*P*<.001, *η_p_*^2^=0.320), humor (*P*<.001, *η_p_*^2^=0.173), and meta-relational talk (*P*=.003, *η_p_*^2^=0.072) in the intended directions, but not for empathy exchanges (*P*=.31, *η_p_*^2^=0.018) or greeting (*P*=.75, *η_p_*^2^=0.006). Figure S4 (a-j) in Multimedia Appendix 1 illustrates the perceived differences between the chatbot personas. Since the manipulation of the interpersonal closeness cues worked as intended, except for empathy exchanges and greeting, the results provide partial support for hypothesis 1.

### No-Choice Group: Effects of Randomly Assigned Chatbot Personas on Outcome Measures

#### Main and Interaction Effects

The MANOVA model specified with chatbot persona, participant gender, and participant age on all outcome variables (ie, interpersonal closeness, affective bond, and intention to use) showed no significant main effects for chatbot persona (*P*=.88) but did so for participant age (Wilks *λ*=0.897, *F*_3,184_=7.010, *P*<.001, *η_p_*^2^=0.103) and participant gender (Wilks *λ*=0.952, *F*_3,184_=3.095, *P*=.03, *η_p_*^2^=0.048). Moreover, a significant interaction effect was found for chatbot persona and participant age (Wilks *λ*=0.887, *F*_9,448_=2.518, *P*=.01, *η_p_*^2^=0.040), showing small effect sizes. Table 2 shows an overview of the MANOVA results.

As the MANOVA discerned significant effects, separate univariate ANOVAs were specified with the same factors as before for each outcome variable following the procedure described in Field [108].

These ANOVAs showed a significant main effect of participant gender on perceived interpersonal closeness (*F*_1,186_=5.923, *P*=.02*, *η*_p_*^2^=0.031) and affective bond (*F*_1,186_=8.081, *P*=.005*, *η*_p_*^2^=0.042), and they showed a significant main effect of participant age only on intention to use (*F*_1,186_=4.528, *P*=.04*, *η*_p_*^2^=0.024).

The interaction effect of participant age and chatbot persona was significant for perceived interpersonal closeness (*F*_1,186_=3.046, *P*=.03*, *η*_p_*^2^=0.047) and affective bond (*F*_1,186_=4.836, *P*=.003*, *η*_p_*^2^=0.072) but not for the intention to use the chatbot (*P*=.10). An excerpt of the ANOVA results for the significant effects is depicted in Table 3. Insignificant effects in the ANOVAs were not further analyzed in the post hoc tests described in the following section.

**Table 2:** Multivariate test results for the specified multivariate analysis of variance model.

Effect			Wilks *λ*		*F* test (*df*)		*P* value		*η_p_^2^*
**Main effect**									
	Chatbot persona	0.976		0.490 (9, 448)		.88		0.008	
	Participant age (<40 years old)	0.897		7.010 (3, 184)		<.001		0.103	
	Participant gender	0.952		3.095 (3, 184)		.03		0.048	
**Two-way interaction effect**									
	Chatbot persona × participant age	0.887		2.518 (9, 448)		.008		0.040	
	Chatbot persona × participant gender	0.969		0.655 (9, 448)		.75		0.011	
	Participant age × participant gender	0.988		0.756 (3, 184)		.52		0.012	
**Three-way interaction effect**									
	Chatbot persona × participant gender × participant age	0.968		0.670 (9, 448)		.74		0.011	

**Table 3:** Analysis of variance (ANOVA) model results for the main effects of participant gender and participant age and two-way interaction between chatbot persona and participant age.

Independent variable^a^	Dependent variable										
	Interpersonal closeness				Affective bond				Intention to use		
	*F* test (*df*)	*P* value	*η_p_^2^*	*F* test (*df*)		*P* value	*η_p_^2^*	*F* test (*df*)		*P* value	*η_p_^2^*
Participant gender	5.923 (1, 186)	.02	0.031	8.081 (1, 186)		.005	0.042	2.170 (1, 186)		.14	0.012
Participant age	2.952 (1, 186)	.09	0.016	1.094 (1, 186)		.30	0.006	4.528 (1, 186)		.04	0.024
Chatbot persona × participant age	3.046 (3, 186)	.03	0.047	4.836 (3, 186)		.003	0.072	2.099 (3, 186)		.10	0.033

^a^Only factors that were significant in the multivariate ANOVA were analyzed in the ANOVAs.

#### Pairwise Comparisons

**Figure 4:**
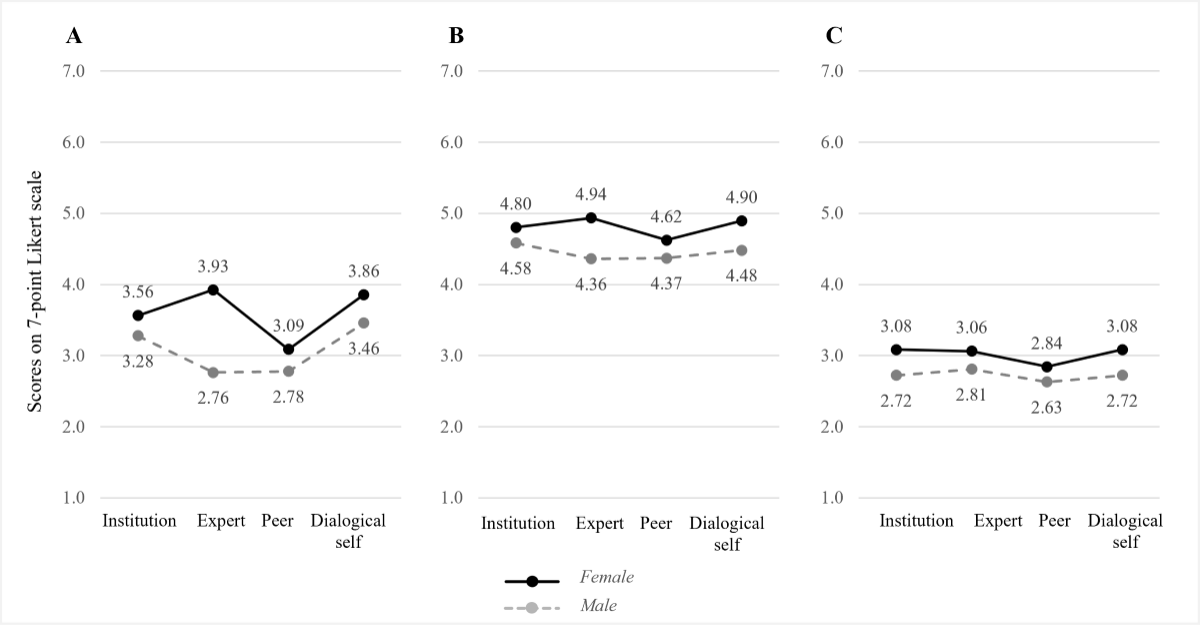
Interaction effects of chatbot personas with participant genders on interpersonal closeness (A), affective bond (B), and intention to use (C).

**Figure 5:**
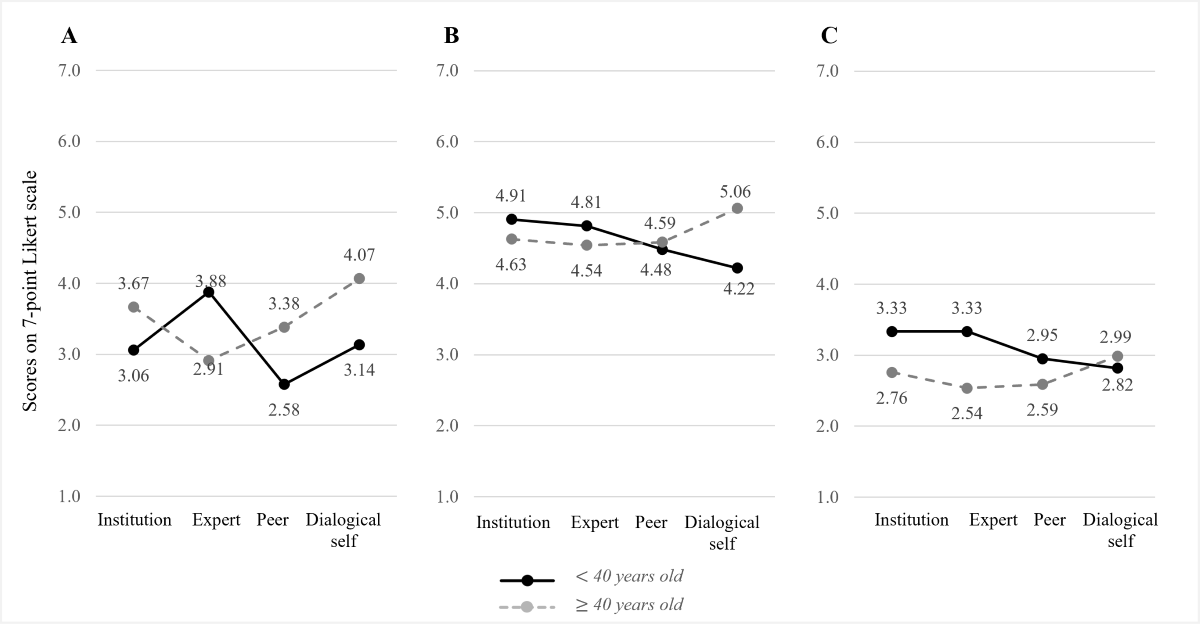
Interaction effects of chatbot personas with participant ages on interpersonal closeness (A), affective bond (B), and intention to use (C).

Pairwise comparisons using Bonferroni correction showed that, regardless of the chatbot persona, superior outcomes were consistently generated for female participants; for instance, female participants reported 0.450 points higher affective bond than male participants (*P*=.005, 95% CI of the difference 0.138-0.762) and 0.633 points higher perceived interpersonal closeness (*P*=.02, 95% CI of the difference 0.120-1.146). Differences in intention to use (*P*=.14) were not statistically significant. Pairwise comparisons between participant age groups (ie, younger than 40 years and 40 years or older) showed that across all chatbot personas, younger participants were more likely to intend to use the chatbot in the future than older participants (mean difference 0.404, SE 0.190; *P*=.04, 95% CI of the difference 0.029-0.778). Figure 4, A to C, and Figure 5, A to C, depict the interaction graphs for all outcome measurements by chatbot persona and participant gender or age, respectively.

Since the interaction effect between chatbot persona and participant age was significant, we inspected the interaction plots (Figure 5, A-C) and conducted two separate simple-effect analyses (1) for each chatbot persona and (2) for each participant age group, respectively.

The analysis of simple effects for each chatbot persona revealed the following significant differences. For the interpersonally closer dialogical-self chatbot, older participants reported significantly higher interpersonal closeness (mean difference 1.354, SE 0.527; *P*=.01) and higher bond scores (mean difference 1.186, SE 0.321; *P*<.001) than younger participants. For the interpersonally more distant expert chatbot, younger participants reported significantly higher intentions to use it than older participants (mean difference 0.805, SE 0.374; *P*=.03).

The analysis of simple effects for each participant age group confirmed that younger participants reported significantly lower affective bond scores (mean difference −1.015, SE 0.363; *P*=.03) for the interpersonally close dialogical-self chatbot compared to the distant institution chatbot. Other differences between the chatbot personas for each participant age group were not statistically significant. Taken together, hypothesis 2 is partially supported, and hypothesis 3 is fully supported.

### Free-Choice Versus No-Choice Group: Effects of Free Choice of the Chatbot Persona on Outcome Measures

In the free-choice group, a chi-square goodness-of-fit test showed that preferences for a chatbot persona were statistically significant (*χ*²_3_=31.4, *P*<.001), with most people (29/49, 59%) choosing the interpersonally close peer chatbot, compared to 8 participants (16%) each choosing the interpersonally more distant institution or expert chatbots. Only 4 participants (8%) chose the extremely close dialogical-self chatbot. Furthermore, cross-gender choice effects were significant in the expert, peer, and dialogical-self versions (*χ*²_3_=4.1, *P*=.04, Cramer *V*=0.318). A total of 84% (16/19) of all female participants chose a female avatar, whereas only 45% (10/22) of male participants chose a male avatar.

To examine the effect of free versus imposed choice, we specified another MANOVA model with choice type, participant gender, and participant age on the same outcome measures as before; however, we only compared perceptions of the peer chatbots, since group sizes of institution, expert, and dialogical-self chatbots in the free-choice group were arguably small.

The MANOVA revealed a significant main effect for choice type (Wilks *λ*=0.802, *F*_3,71_=5.856, *P*=.001, *η_p_*^2^=0.198), but no significant main or interaction effects for or with participant gender (*P*=.55) or age (*P*=.21), respectively. Table S5 in Multimedia Appendix 1 provides an overview of the MANOVA results. Subsequently conducted univariate ANOVAs specified with the same factors on each outcome variable confirmed the significant main effect of choice type on all outcome variables. For an overview, see Table S6 in Multimedia Appendix 1. Post hoc pairwise comparisons revealed that participants who had the option to freely choose the peer chatbot evaluated it consistently better than participants who had been imposed to interact with it; for example, affective bond with the peer chatbot had a mean score of 5.28 (SD 0.89) in the free-choice group compared to a mean score of 4.54 (SD 1.10) in the no-choice group. Similarly, participants who had freely chosen the chatbot felt closer to the chatbot than those who did not choose it (mean difference 1.613, SE 0.466; *P*=.001), and they were more likely to intend to use it (mean difference 1.294, SE 0.324; *P*<.001). Taken together, hypothesis 4 is supported.

## Discussion

### Principal Findings

Our design codebook for chatbots with different social roles provides a novel approach to design chatbots along an interpersonal closeness continuum that is inspired by clients’ encounters with different social health care roles in their client journey: health care institutions, medical experts, peers, and themselves (ie, hypothesis 1). The results from the web-based experiment suggest that a chatbot’s impersonated social role affects users’ perception and the development of an affective bond contingent on users’ demographic profiles. Since the main effect of the chatbot personas on the outcome measures was not significant, our study strengthens the recommendation that it is necessary to take into account user-specific factors before developing generic one-fits-all designs (ie, hypothesis 3).

Specifically, we found a significant age difference in chatbot assessments, this is, older participants rated the dialogical-self character in significantly more positive ways than younger participants, and younger participants consistently preferred the expert character. We assume that whereas younger participants valued guidance from a more distant “authority role” [27], older users seemed more intrigued by the idea to find guidance in themselves. Since older age is also associated with a greater number of life experiences and, consequently, more knowledge and judgment about life and ways of planning, managing, and understanding life [112], older participants might have had more trust in their expertise than younger participants. Thus, older participants’ positive reception of the dialogical-self chatbot might generally point to an untapped potential of mimicking self-talk and inner dialogue by health care chatbots. Changing the way people think about and talk to themselves is a fundamental principle of cognitive behavioral therapy [113], an approach commonly implemented in many web-based [114] and chatbot-based mental health interventions [115]. Making a chatbot explicitly mimic one’s inner voice represents an innovative approach to help clients experience how positive self-talk can look and feel.

Lastly, our study shows that giving individuals the option to choose between a range of presented chatbot characters can have an effect on their chatbot preferences (ie, hypothesis 4). Specifically, in our study, we found that free choice significantly improved participants’ perceptions of the peer chatbot. This strengthens the recommendation that it is worth the extra effort to integrate even simple customization and personalization options [71,75]. Necessary user information, such as gender and age, could be easily elicited during onboarding based on responses to survey questions posed by the chatbot.

### Limitations and Future Research Directions

This work has several limitations that point to future research directions upon which researchers can seize.

First, despite the scientific rigor employed to develop four distinct chatbot personas by manipulating a holistic set of interpersonal closeness cues derived from previous research, the conceptualized social roles can only be considered as design archetypes. Future experiments could examine nuances of the conceptualized social roles as well as which design cues are most relevant for clients’ perceptions of a social role (research direction 1). Furthermore, our scenario covered a health care chatbot providing a particular type of health intervention. Whereas social role dynamics in a provider-client relationship in lifestyle interventions are likely similar in their essence to other health interventions (ie, highly sensitive, emotional, personal, and interpersonally intense), future research could explicitly compare differences in distinct health contexts, for example, between health care chatbots for different chronic diseases and those for preventive care (research direction 2).

Second, another limitation of our study concerns its limitation to German-speaking people ranging in age from 18 to 65 years only. Digital interventions addressing younger or older clients beyond this age range (eg, older people suffering from dementia [76] or younger clients with child obesity [116]) are likely to require different designs, content, and functionalities of the chatbot (research direction 3). For instance, the interpersonal relationship of an older client with cancer with a peer chatbot is likely to be of another kind than the relationship of a pregnant woman with a “pregnant” peer chatbot. Thus, depending on the health care setting, disease, or the specific stage of a disease [76], future research could explore further interesting specialized social roles that could be impersonated by a health care chatbot, such as professionals (ie, nurses, midwives, physiotherapists, pharmacists, etc) and health care social workers, which could help with alleviating the financial, social, and psychological burden of chronic disease. Other social roles that could be impersonated by a chatbot could be those of family members, for example, in digital pediatric or partner health interventions (research direction 4).

Third, another consideration concerns the length of the prospective client-chatbot relationship. In our experiment, participants were interacting with a chatbot prototype that simulated three nonconsecutive “days” of a fictitious health care intervention for only about 16 minutes in a time-lapsed manner. However, relationships develop and change in depth and breadth over time [95]. Therefore, the effects that we detected likely reflected participants’ initial affect levels toward the chatbot, which would explain the smaller effect sizes as well. Future research could examine the impact of a chatbot’s social role in a fully operational prototype (research direction 5) to investigate changes in users’ evaluations of the social role over time (research direction 6) or the choice of appropriate social roles at the various stages of the client journey (research direction 7). Future research could also examine the impact of a chatbot’s social role, ultimately, on actual therapeutic outcomes (research direction 8), depending on the health care setting. For instance, similar to human-human interactions, some social roles might be more appropriate for communicating a life-threatening diagnosis, whereas another social role might be more appropriate for helping clients monitor a specific vital parameter every day. Eventually, our experimental design only allowed us to measure behavioral *intentions,* which do not necessarily translate into behavior (cf, intention-behavior gap [117]). Future field experiments should examine how the design of the chatbot actually affects, for instance, the likelihood to share sensitive personal health information (research direction 9).

Fourth, even if one result of our research was to match every client with their personalized perfect chatbot, or at least to provide enough freedom of choice between a couple of chatbot characters to improve clients’ evaluation of a chatbot, the development of multiple chatbots delivering the same intervention adds complexity to the development of digital health interventions, thereby requiring more financial resources and time. Future research should seek to explore the optimal levels of personalization, customization, and choice options by contrasting them with development resources (research direction 10). In a similar vein, future researchers should also investigate additional individual characteristics as potential control variables, such as people’s tendency to anthropomorphize artificial objects or their perceived creepiness when interacting with anthropomorphized technology (research direction 11) [69].

Finally, this study indicated that there were substantial age effects in the perception of different conversational agents within a cross-sectional design. In the case that these age effects can be replicated, future research investigating longitudinal designs is needed (research direction 12) to better understand whether such potential effects are impacted by particular cohorts and particular ages in the context of personality change (eg, Marsh et al [118]) and other digital health interventions.

### Comparison With Prior Work

To the best of our knowledge, this is the first study to configure and compare health care chatbots with different impersonated social roles common in health care settings. By studying the impact of chatbots’ social roles and integrating knowledge from the social role theory, this work extends prior research that has investigated the effects of relational cues [65], and it takes on prior calls for research to consider an agent’s impersonated social role as an important factor in user-chatbot relationships [27,119]. This research thus contributes to the understanding of the design of health care chatbots by defining how a set of interpersonal closeness cues manifest the social role of a chatbot, as well as by determining how these social roles affect clients’ experiences and the development of an affective bond with the chatbot.

Furthermore, our study contributes to prior research on the importance of personal characteristics in people’s decisions to interact with chatbots [68] and shows that user perceptions of specific social roles depend on a person’s demographic profile, namely an individuals’ age and gender, and whether individuals could freely choose the chatbot persona or not.

### Conclusions

Since chatbots are becoming increasingly prevalent in clients’ health care service experiences, health care providers’ success depends on their ability to design chatbots effectively. Especially in the context of chronic diseases, where digital health interventions are aimed at accompanying clients for years, it is still open as to which design choices promote the development of a strong affective bond between chatbot and client. To this end, we developed a codebook that allows researchers and practitioners to systematically design health care chatbots with specific social roles common in health care settings, and we explored whether or how these social roles affect the development of an affective bond between the user and the chatbot. Overall, our results suggest that positive effects can come from customizing the chatbot persona to easily accessible user characteristics, such as age and gender, or from allowing clients to choose the social role they feel they need most. Future work is required to investigate role dynamics in client-chatbot relationships during longer-term interventions.

## Appendix

Multimedia Appendix 1 Supplementary material.

## Abbreviations

ANOVA: analysis of variance
CDHI: Centre for Digital Health Interventions
ECIS 2018: European Conference on Information Systems 2018
MANOVA: multivariate analysis of variance
T/V: *tu/vos*
