# Supplementary material for: The Effects of Health Care Chatbot Personas With Different Social Roles on the Client-Chatbot Bond and Usage Intentions: Development of a Design Codebook and Web-Based Study
Source: J Med Internet Res. 2022 Apr 27;24(4):e32630. doi: 10.2196/32630 (PMC9096656; doi:10.2196/32630)
Supplement: Multimedia Appendix 1 [file jmir_v24i4e32630_app1.pdf]

The Effects of Health Care Chatbot Personas With Different Social Roles on the Client-Chatbot Bond and Usage Intentions: Development of a Design Codebook and Web-Based Study

Supplemental Material

Tables **2**

Table S1. Detailed overview and analysis of previously considered design cues in text-based conversational agent studies 2

Table S2. Detailed overview and analysis of previously considered anthropomorphic cues in embodied conversational agent studies 3

Table S3: List of measures 5

Table S4: List of manipulation check items 6

Table S5. Multivariate test results for MANOVA with choice type, participant age and participant gender 7

Table S6. Univariate test results from ANOVAs for main effect of choice type 8

Figures **9**

Figure S1. Introductory statement to experimental stimuli (Institution) 9

Figure S2. Self-selection question for free-choice group 10

Figure S3. Screenshot of the prototype app embedded on a webpage with appetize.io 11

Figure S4. Manipulation check results: Interaction graphs 12

References **13**

Tables

Table S1. Detailed overview and analysis of previously considered design cues in text-based conversational agent studies

Reference	CA Type	Investigated design cue(s)	Outcome measurements	Verbal Relational Content	Verbal Style	Non-verbal style	Visual
Carfora, Bertolotti, & Catellani, 2019	**TCA**	▪ Informational vs. emotional messages	▪ Meat consumption, attitude, intention, anticipated regret	✓	-	-	-
Liu & Sundar, 2018	**TCA**	▪ Three types of empathic expression	▪ Eeriness, likeability, intelligence, novelty	✓	-	-	-
M. Lee et al., 2019	**TCA**	▪ Caregiving vs. care receiving behaviors of a chatbot	▪ Self-compassion, 'Inclusion of Other in the Self'-scale, error rate, total word count	✓	-	-	-
Y.-C. Lee, Yamashita, Huang, & Fu, 2020	**TCA**	▪ None, low and high self-disclosure	▪ Word count qualitatively observed self-disclosure level	✓	-	-	-
Araujo, 2018	**TCA**	▪ Language style, name, framing used to introduce the chatbot	▪ Social presence, mindful and mindless anthropomorphism	✓	✓	-	-
Shi et al., 2020	**TCA**	▪ Human name vs. chatbot name, personal vs. non-personal inquiry strategy	▪ Donation probability, partner impression, conversation quality, engagement	✓	✓	-	-
Ho & Hancock, 2019	**TCA**	▪ Emotional vs. factual disclosures, chatbot vs. person identity	▪ Warmth, self-affirmation, understanding, disclosure intimacy, cognitive reappraisal	✓	✓	-	-
Chattaraman, Kwon, Gilbert, & Ross, 2019	**TCA**	▪ Task vs. social-oriented communication style, text vs voice, low vs. high internet competency	▪ Social, functional, and behavioral intent outcomes, trust, ease of use, self-efficacy	✓	✓	-	-
S. Lee, Lee, & Sah, 2019	**TCA**	▪ Paralinguistic and backchanneling cues	▪ Mind perception, co-presence, closeness, intention to use	-	✓	-	-
Westerman, Cross, & Lindmark, 2019	**TCA**	▪ Typos and capitalized words	▪ Perceived humanness, task attraction, anthropomorphism, social attraction	-	✓	-	-
Fadhil, Schiavo, Wang, & Yilma, 2018	**TCA**	▪ Emojis	▪ Enjoyment, attitude, confidence	-	-	✓	-
Beattie, Edwards, & Edwards, 2020	**TCA**	▪ Emojis	▪ Interpersonal attractiveness, competence, source credibility	-	-	✓	-
Candello, Pinhanez, & Figueiredo, 2017	**TCA**	▪ Typeface	▪ Qualitatively observed levels of perceived humanness and others	-	-	✓	-
McDonnell & Baxter, 2019	**TCA**	▪ Gender-stereotypical visual appearance	▪ Satisfaction, gender-stereotypical perception	-	-	-	✓
Go & Sundar, 2019	**TCA**	▪ Visual anthropomorphism, identity cue, message interactivity	▪ Expertise, friendliness, intelligence, competence, behavioral intentions	✓	✓	-	✓
**Our work**	**TCA**	▪ Typical healthcare social role	▪ Affective bond, interpersonal closeness, intention to use	✓	✓	✓	✓
**Total ***	**15***			**9***	**7***	**3***	**2***
Relative Share				*60.0%*	*46.7%*	*20.0%*	*13.3%*

*Note. All articles included were selected from the systematic literature review on chatbots by Rapp, Curti, and Boldi (2021); CA = Conversational Agent; TCA = Text-based Conversational Agent (= chatbot); ✓ = Type of design cue was considered in this article's study(ies); * = Without this paper*

Table S2. Detailed overview and analysis of previously considered anthropomorphic cues in embodied conversational agent studies

Reference	CA Type	Investigated design cue(s)	Outcome measurements	Verbal Relational Content	Verbal Style	Non-verbal style	Visual
Parmar, Olafsson, Utami, & Bickmore, 2018	**ECA**	▪ Role-appropriated business attire	▪ Perceived professionalism, trustworthiness, persuasiveness	-	-	-	✓
Robertson et al., 2016	**ECA**	▪ Photographic vs. highly stylized renderings	▪ Ease of use, satisfaction, helpfulness	-	-	-	✓
Schmeil & Suggs, 2014	**ECA**	▪ Body shape	▪ Intention to change behavior	-	-	-	✓
Skalski & Tamborini, 2007	**ECA**	▪ Interactivity, attractiveness	▪ Social presence, message processing, attitudes, behavioral intentions	-	-	-	✓
Van Vugt, Konijn, Hoorn, & Veldhuis, 2006	**ECA**	▪ Appearance (similar vs. dissimilar, slim vs. fat)	▪ Helpfulness, preference, usage intentions	-	-	-	✓
Van Wissen, Vinkers, & van Halteren, 2016	**ECA**	▪ Appearance (age, stylized vs. photo)	▪ Familiarity, usefulness	-	-	-	✓
Alsharbi & Richards, 2017	**ECA**	▪ Visual appearance	▪ Preference, rapport	-	-	-	✓
Forlizzi, Zimmerman, Mancuso, & Kwak, 2007	**ECA**	▪ Visual appearance (age, gender)	▪ Preference	-	-	-	✓
Nguyen & Masthoff, 2007	**ECA**	▪ Visual appearance (age, gender)	▪ Source credibility	-	-	-	✓
Ring, Utami, & Bickmore, 2014	**ECA**	▪ Character proportion, rendering style	▪ Trustworthiness, eeriness, desire to continue interact, etc.	-	-	-	✓
Zhou, Bickmore, Paasche-Orlow, & Jack, 2014	**ECA**	▪ Socio demographic appearance (racial concordance, black vs. white)	▪ Liking, trust, desire to continue, Working alliance	-	-	-	✓
Nguyen & Masthoff, 2009	**ECA**	▪ Modality, animated vs. no visual, empathy vs. non empathy	▪ Affective state, task performance, credibility, enjoyment, trustworthiness	✓	-	-	✓
Kang & Gratch, 2011	**ECA**	▪ Self-disclosure	▪ Co-presence, social attraction	✓	-	-	-
Olafsson, Kimani, Asadi, & Bickmore, 2017	**ECA**	▪ Acoustic manipulation of speech, discussed topic (nutrition vs. exercise)	▪ Preference, engagement	-	✓	-	-
Yin, Bickmore, & Cortés, 2010	**ECA**	▪ Appearance and language (English/ Spanish) of the chatbot, cultural background of the user	▪ Persuasiveness, cultural and linguistic congruity	-	✓	-	✓
Bickmore & Picard, 2005	**ECA**	▪ No agent vs. relational vs. non-relational	▪ Respect, desire to continue interacting, trust, likeability,	✓	✓	-	✓
Bickmore, Gruber, & Picard, 2005	**ECA**	▪ Web form only, relational vs. non-relational	▪ Working alliance, liking, desire to continue interacting, usefulness	✓	✓	-	✓

(continued on next page)

Table S2. continued

Reference	CA Type	Investigated design cue(s)	Outcome measurements	Verbal Relational Content	Verbal Style	Non-verbal style	Visual
Zhou, Zhang, & Bickmore, 2017	**ECA**	▪ Verbal (power distance, small talk) and visual (avatar) cultural adaptation	▪ Satisfaction, trust	✓	✓	-	✓
Bickmore & Schulman, 2007	**ECA**	▪ Expressive vs. empathic chatbot	▪ Affective state, satisfaction, intention to use, likeability	✓	✓	-	-
Bickmore, Schulman, & Yin, 2009	**ECA**	▪ 1st person vs. 3rd person narrative	▪ Enjoyment, engagement, honesty	✓	✓	-	-
Bickmore, Schulman, & Yin, 2010	**ECA**	▪ Content variability, first person narrative vs. 3rd person narrative	▪ Desire to continue interaction, perceived repetitiveness,	✓	✓	-	-
Amini, Lisetti, Yasavur, & Rishe, 2013	**ECA**	▪ Empathic vs. non empathic facial expression	▪ Enjoyment, engaging, likeability,	✓	✓	✓	-
Frost, Boukris, & Roelofsma, 2012	**ECA**	▪ Movement, interactivity, compliment, personal feedback	▪ Intrinsic motivation, self-efficacy, parasocial interaction	✓	✓	✓	-
Bickmore & Picard, 2004	**ECA**	▪ Relational vs. non-relational verbal and facial expression	▪ Affective bond, desire to continue interacting,	✓	✓	✓	-
Amini, Lisetti, & Yasavur, 2014	**ECA**	▪ Empathic vs. non empathic facial expression vs. text-only	▪ Trust, intention to use, perceived usefulness, etc.	✓	✓	✓	✓
Lisetti, Amini, Yasavur, & Rishe, 2013	**ECA**	▪ Empathic vs. non empathic facial expression vs. text-only	▪ Acceptance, enjoyment, intention to use	✓	✓	✓	✓
Bickmore & Ring, 2010	**ECA**	▪ Speech vs textual output, control over verbal and nonverbal behavior	▪ Satisfaction, ease of use, helpfulness	-	✓	✓	-
Malhotra, Hoey, König, & Vuuren, 2016	**ECA**	▪ Appearance (gender), empathic facial expressions	▪ Evaluation, potency, activity	-	-	✓	✓
Silverman et al., 2001	**ECA**	▪ Emotive vs non-emotive multimedia version	▪ Memory retention, intention to call 9-1-1	-	-	✓	✓
Creed & Beale, 2012	**ECA**	▪ Emotional vs. non-emotional facial expressions	▪ Caring, liking, intention to use	-	-	✓	-
Creed, Beale, & Cowan, 2015	**ECA**	▪ Emotional vs. non-emotional facial expressions	▪ Working alliance, drop-out rates, fruit consumption	-	-	✓	-
Grillon & Thalmann, 2008	**ECA**	▪ Facial, gaze, hand, body gestures	▪ Interest, engagement, friendliness, normality	-	-	✓	-
**Total**	**32**			**13**	**14**	**11**	**20**
Relative Share				*40.6%*	*43.8%*	*34.4%*	*62.5%*

Note. All articles included were selected from the systematic literature review on chatbots by ter Stal, Kramer, Tabak, op den Akker, and Hermens (2020); CA = Conversational Agent; ECA = Embodied Conversational Agent; ✓ = Type of design cue was considered in this article's study(ies)

Table S3: List of measures

**Theme**	**Construct**	*Reference*
**Working alliance inventory** for technology-based health interventions	**Attachment Bond**<br>*7-point Likert, 1 = fully disagree to 7 = fully agree*<br>I think $chatbot* liked me.<br>$chatbot and I respected each other.<br>I feel that $chatbot appreciated me.<br>I feel like $chatbot cared about me even when I did something that $chatbot did not approve of.<br>I felt uncomfortable with $chatbot. *[reverse coded]*<br>$chatbot and I understood each other.<br>I think $chatbot was genuinely concerned for my well-being.<br>I feel that $chatbot was not completely honest about his/her/its feelings toward me. *[reverse coded]*<br>I was confident in $chatbot's ability to help me.<br>$chatbot and I trusted each other.<br>The relationship with $chatbot was very important to me.<br>I had the feeling that if I said or did the wrong things $chatbot would stop working with. *[reverse coded]*	*(Kiluk, Serafini, Frankforter, Nich, & Carroll, 2014)*
**Perceived interpersonal closeness**	**Inclusion of Others in Self Scale (IOS)**<br>*7-point pictorial scale, 1 = extremely distant to 7 = extremely close*<br>"With this question, we would like you to assess which of the following pairs of circles best describes your relationship with $chatbot. By choosing a pair of circles, you indicate how strongly you feel connected to $chatbot.<br><br>*In the images, "x" represents $chatbot, so please imagine "$chatbot" in the circles.*<br>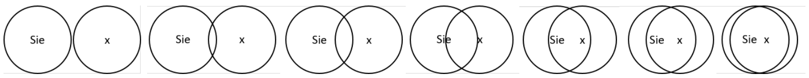<br>*1 2 3 4 5 6 7*<br>*x = $chatbot*	*(Aron, Aron, & Smollan, 1992)*
**Technology acceptance model (TAM)**	**Intention to Use**<br>*5-point Likert, 1 = fully disagree to 5 = fully agree)*<br>I would chat regularly with $chatbot if I had access to it.<br>I would like to chat with $chatbot about other topics if I had access to it.<br>I could imagine to increase my interactions with $chatbot in the next year.	(Wixom & Todd, 2005)
**Socio-demographics**	What is your **gender**? (female, male, other);<br>How **old** are you? (free, text entry);<br>Please indicate your **native language.** (German, French, Other)	Own items

Note. **The variable "$chatbot" displayed the chatbot's name (i.e., INSTITUTION: "(der) Persönlichkeitscoach"; EXPERT: "Herr/Frau Dr.Change", PEER: "Mila/Milo/Mira/Miro"; DIALOGICALSELF: "MySelfCoach" respectively); items translated into English by the authors for this paper*

Table S4: List of manipulation check items

Interpersonal Closeness Cue	Item
**Verbal cues**	***$chatbot* …***
Form of address	... talked to me in a personal way.
Jargon	... used professional jargon.
T/V distinction	... used the T form [Du] with me.
**Quasi non-verbal cues**	
Emoji	... used emojis.
**Relational cues**	
Greeting	… greeted me in a friendly manner.
Social dialogue	... chitchatted with me about things beyond the content of the program.
Meta-relational talk	... talked with me about our relationship.
Self-disclosure	... told something about himself and his own experiences.
Empathy	... responded emphatically to me and my answers.
Humor	... made jokes.

*Note. Own items; all measured on 5-point Likert scales from 1 = doesn't apply at all to 5 = fully applies; pre-tested among five experts and 22 participants from a convenience sample. *The variable "$chatbot" displayed the chatbot's name (i.e., INSTITUTION: "(der) Persönlichkeitscoach"; EXPERT: "Herr/Frau Dr.Change", PEER: "Mila/Milo/Mira/Miro"; DIALOGICALSELF: "MySelfCoach" respectively); items translated into English by the authors for this paper*

Table S5. Multivariate test results for MANOVA with choice type, participant age and participant gender

**Effect**	***df***	***Wilks' λ***	***F***	***p***	η_p^2
Main effect					
**Choice type**	3	.802	5.856	**.001**	.198
Participant age (< 40 years old = 1)	3	.938	1.568	.205	.062
Participant gender	3	.971	0.706	.551	.029
Two-way interaction effect					
Choice type * participant age	3	.958	1.040	.380	.042
Choice type * participant gender	3	.994	0.131	.941	.006
Participant age * participant gender	3	.972	0.675	.570	.028
Three-way interaction effect					
Choice type * participant gender * participant age	3	.983	.413	.744	.017

Note. Significant values at ***p < .05*** *are in* ***boldface***.

Table S6. Univariate test results from ANOVAs for main effect of choice type

Dependent variables	Interpersonal closeness			Affective bond			Intention to use		
Independent variables	***F***	***p***	η_p^2	***F***	***p***	η_p^2	***F***	***p***	η_p^2
Choice type	11.963	**.001**	.141	9.634	**.003**	.117	15.964	**.000**	.179

Note. Significant values at ***p < .05*** *are in* ***boldface.*** *Only factors that were significant in the MANOVA were analyzed in the ANOVAs.*

Figures

Figure S1. Introductory statement to experimental stimuli (Institution)

Welcome to the main part of our study

During the following five to ten minutes, you will have the opportunity to chat with the virtual PersonalityCoach, $chatbot, developed specially for this study.

In the course of the conversation, you will get to know each other and be able to talk for some time. The PersonalityCoach will introduce you to exemplary exercises from a PersonalityChange program but you will not have to perform them or evaluate their content. We are only interested in your first impression of the conversation with it.

PersonalityCoach

UNIVERSITAS TURICENSIS MDCCC XXXIII

"The PersonalityCoach is a 'digital coach' developed by researchers, experts and psychotherapists from the University of Zurich which has been equipped with various skills based on the latest findings from many years of research on 'personality development'

The PersonalityCoach has been programmed to represent the Psychological Institute of the University of Zurich, which enjoys a very good reputation worldwide in the field of personality research.

*Note. *The variable "$chatbot" displayed the chatbot's name (i.e., Institution chatbot: "(der) PersönlichkeitsCoach"; Expert chatbot: "Herr/Frau Dr.Change"; Peer chatbot: "Mila/Milo/Mira/Miro"; Dialogical-self chatbot: "MySelfCoach" respectively); Equivalently, the avatar/icon was exchanged based on the treatment condition; Figure translated into English by the authors*

Figure S2. Self-selection question for free-choice group

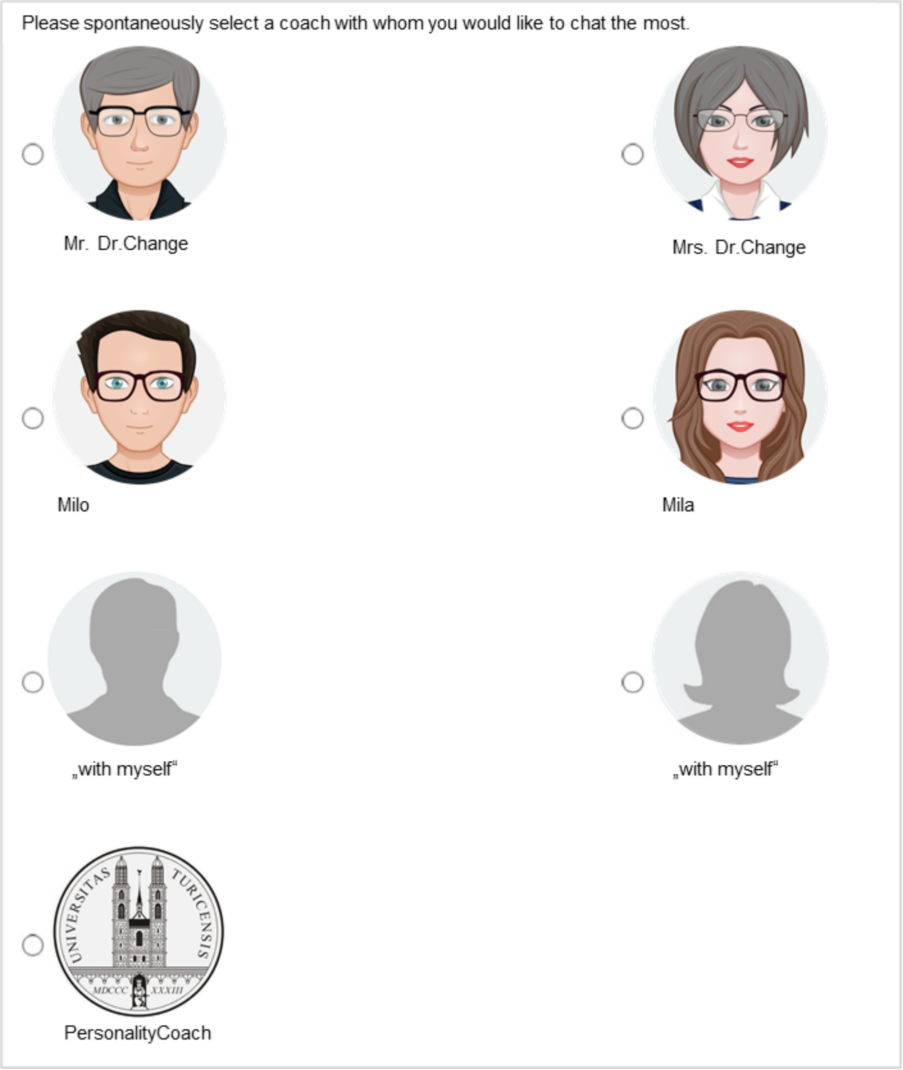

Figure S3. Screenshot of the prototype app embedded on a webpage with appetize.io

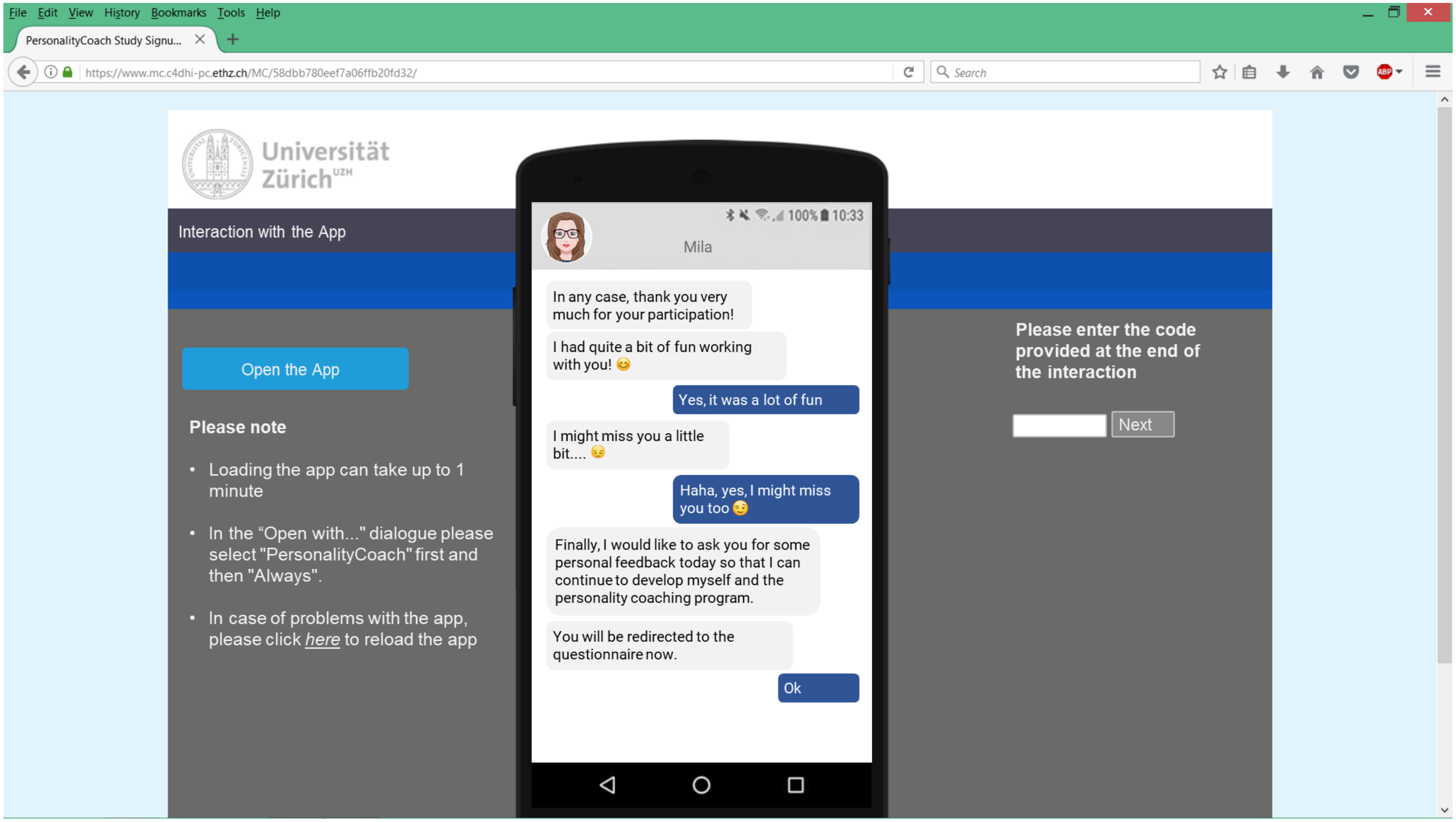

Note. *The study has been conducted in German. This screenshot has been translated into English by the authors for this paper.*

Figure S4. Manipulation check results: Interaction graphs

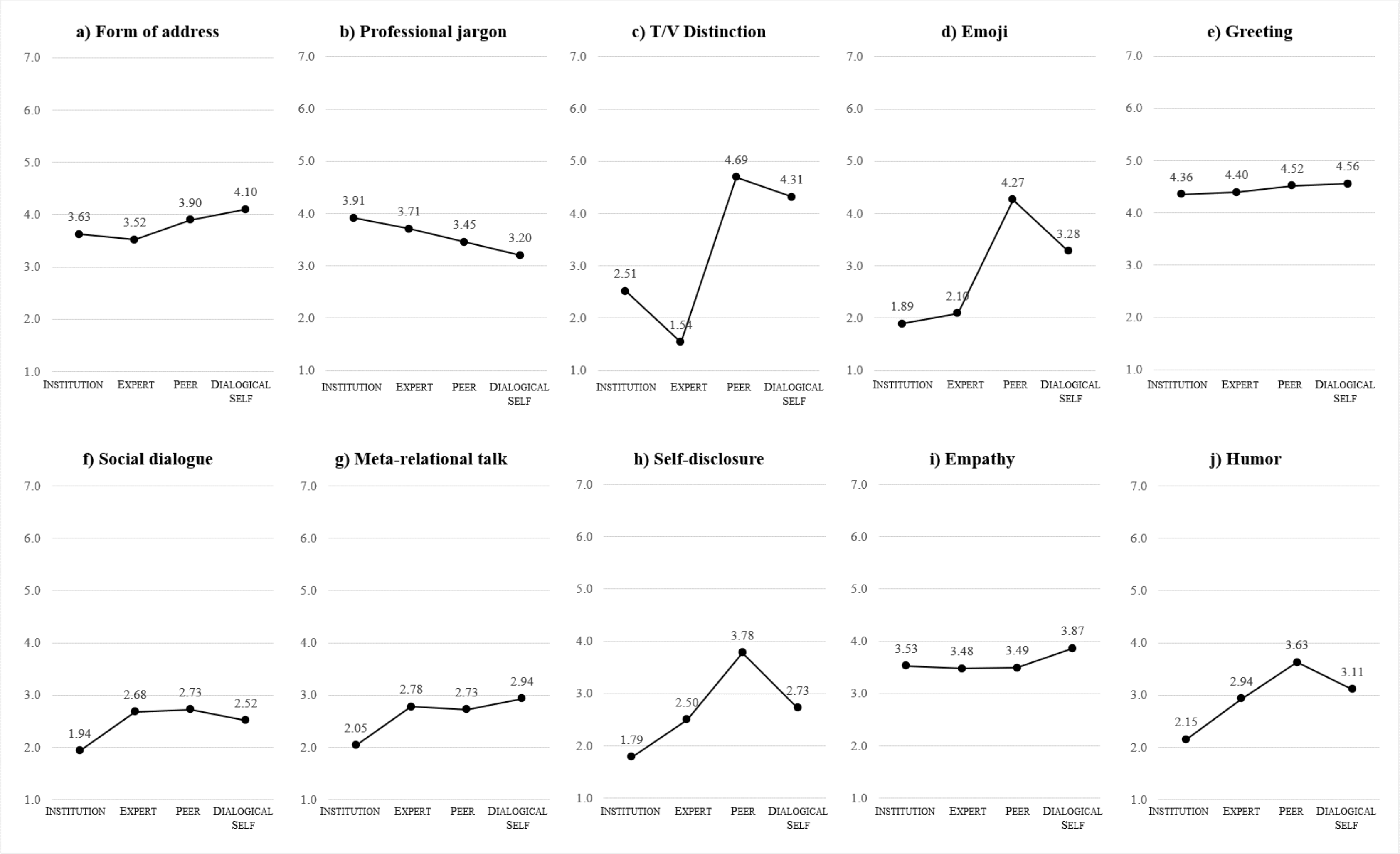

References

Alsharbi, B., & Richards, D. (2017)**.** Using virtual reality technology to improve reality for young people with chronic health conditions. *the 9th International Conference.* Proceedings of the 9th International Conference on Computer and Automation Engineering (ICCAE '17), 11–15. doi:10.1145/3057039.3057080

Amini, R., Lisetti, C., & Yasavur, U. (2014)**.** Emotionally responsive virtual counselor for behavior-change health interventions. In Hutchison, D. et al. (Eds.). Proceedings of the 9th International Conference on Design Science Research in Information Systems and Technology (DESRIST '14), *Lecture Notes in Computer Science*, 433–437. doi:10.1007/978-3-319-06701-8_40

Amini, R., Lisetti, C., Yasavur, U., & Rishe, N. (2013)**.** On-demand virtual health counselor for delivering behavior-change health interventions. *2013 IEEE International Conference on Healthcare Informatics (ICHI).* Proceedings of the IEEE International Conference on Healthcare Informatics (ICHI '13), 46–55. doi:10.1109/ICHI.2013.13

Araujo, T. (2018)**.** Living up to the chatbot hype: The influence of anthropomorphic design cues and communicative agency framing on conversational agent and company perceptions. *Computers in Human Behavior*, *85*, 183–189. doi:10.1016/j.chb.2018.03.051

Aron, A., Aron, E. N., & Smollan, D. (1992)**.** Inclusion of other in the self scale and the structure of interpersonal closeness. *Journal of Personality and Social Psychology*, *63*(4), 596–612. doi:10.1037//0022-3514.63.4.596

Beattie, A., Edwards, A. P., & Edwards, C. (2020)**.** A bot and a smile: interpersonal impressions of chatbots and humans using emoji in computer-mediated communication. *Communication Studies*, *71*(3), 409–427. doi:10.1080/10510974.2020.1725082

Bickmore, T. W., Gruber, A., & Picard, R. W. (2005)**.** Establishing the computer-patient working alliance in automated health behavior change interventions. *Patient Education and Counseling*, *59*(1), 21–30. doi:10.1016/j.pec.2004.09.008

Bickmore, T. W., & Picard, R. W. (2004)**.** Towards caring machines. In Dykstra-Erickson, E. et al. (Eds.). Extended Abstract on Human Factors in Computing Systems (CHI EA '04), 1489–1492. doi:10.1145/985921.986097

Bickmore, T. W., & Picard, R. W. (2005)**.** Establishing and maintaining long-term human-computer relationships. *ACM Transactions on Computer-Human Interaction*, *12*(2), 293–327. doi:10.1145/1067860.1067867

Bickmore, T. W., & Ring, L. (2010)**.** Making it personal: End-user authoring of health narratives delivered by virtual agents. In Allbeck, J. (Ed.). Proceedings of the 10th International Conference on Intelligent Virtual Agents (IVA '10), *Lecture Notes in Computer Science, 6356*, 399–405. doi:10.1007/978-3-642-15892-6_43

Bickmore, T. W., & Schulman, D. (2007)**.** Practical approaches to comforting users with relational agents. In Rosson, M. B. (Ed.). Extended Abstracts on Human Factors in Computing Systems (CHI EA '07), 2291–2296. doi:10.1145/1240866.1240996

Bickmore, T. W., Schulman, D., & Yin, L. (2009)**.** Engagement vs. Deceit: Virtual humans with human autobiographies. In Ruttkay, Z. et al. (Eds.). Proceedings of the 9th International Conference on Intelligent Virtual Agents (IVA '09), *5773*, 6–19. doi:10.1007/978-3-642-04380-2_4

Bickmore, T. W., Schulman, D., & Yin, L. (2010)**.** Maintaining engagement in long-term interventions with relational agents. *Applied Artificial Intelligence*, *24*(6), 648–666. doi:10.1080/08839514.2010.492259

Candello, H., Pinhanez, C., & Figueiredo, F. (2017)**.** Typefaces and the perception of humanness in natural language chatbots. *CHI '17: CHI Conference on Human Factors in Computing Systems.* In Mark, G. (Ed.). Proceedings of the Conference on Human Factors in Computing Systems (CHI '17), 3476–3487. doi:10.1145/3025453.3025919

Carfora, V., Bertolotti, M., & Catellani, P. (2019)**.** Informational and emotional daily messages to reduce red and processed meat consumption. *Appetite*, *141*, 104331. doi:10.1016/j.appet.2019.104331

Chattaraman, V., Kwon, W.-S., Gilbert, J. E., & Ross, K. (2019)**.** Should ai-based, conversational digital assistants employ social- or task-oriented interaction style? A task-competency and reciprocity perspective for older adults. *Computers in Human Behavior*, *90*, 315–330. doi:10.1016/j.chb.2018.08.048

Creed, C., & Beale, R. (2012)**.** User interactions with an affective nutritional coach. *Supportive Interaction: Computer Interventions for Mental Health*, *24*(5), 339–350. doi:10.1016/j.intcom.2012.05.004

Creed, C., Beale, R., & Cowan, B. (2015)**.** The impact of an embodied agent's emotional expressions over multiple interactions. *Supportive Interaction: Computer Interventions for Mental Health*, *27*(2), 172–188. doi:10.1093/iwc/iwt064

Fadhil, A. S., Schiavo, G., Wang, Y., & Yilma, B. A. (2018)**.** The effect of emojis when interacting with conversational interface assisted health coaching system. *PervasiveHealth '18: 12th EAI International Conference on Pervasive Computing Technologies for Healthcare.* In Minsky, N. et al. (Eds.). Proceedings of the 12th EAI International Conference on Pervasive Computing Technologies for Healthcare, 378–383. doi:10.1145/3240925.3240965

Forlizzi, J., Zimmerman, J., Mancuso, V., & Kwak, S. (2007)**.** How interface agents affect interaction between humans and computers. *the 2007 conference.* In Koskinen, I. et al. (Eds.). Proceedings of the Conference Designing Pleasurable Products and Interfaces (DPPI '07), 209–221. doi:10.1145/1314161.1314180

Frost, J., Boukris, N., & Roelofsma, P. (2012)**.** We like to move it move it! Motivation and parasocial interaction. *CHI '12: CHI Conference on Human Factors in Computing Systems.* In Konstan, J. A. (Ed.). Proceedings of the 30th Conference on Human Factors in Computing Systems (CHI '12), 2465–2470. doi:10.1145/2212776.2223820

Go, E., & Sundar, S. S. (2019)**.** Humanizing chatbots: The effects of visual, identity and conversational cues on humanness perceptions. *Computers in Human Behavior*, *97*, 304–316. doi:10.1016/j.chb.2019.01.020

Grillon, H., & Thalmann, D. (2008)**.** Eye contact as trigger for modification of virtual character behavior. *2008 Virtual Rehabilitation.* Virtual Rehabilitation, 2008, 205–211. doi:10.1109/ICVR.2008.4625161

Ho, S. M., & Hancock, J. T. (2019)**.** Context in a bottle: language-action cues in spontaneous computer-mediated deception. *Computers in Human Behavior*, *91*, 33–41. doi:10.1016/j.chb.2018.09.008

Kang, S.-H., & Gratch, J. (2011)**.** People like virtual counselors that highly-disclose about themselves. *Studies in Health Technology and Informatics*, *167*, 143–148. doi:10.3233/978-1-60750-766-6-143

Kiluk, B. D., Serafini, K., Frankforter, T., Nich, C., & Carroll, K. M. (2014)**.** Only connect: The working alliance in computer-based cognitive behavioral therapy. *Behaviour Research and Therapy*, *63*, 139–146. doi:10.1016/j.brat.2014.10.003

Lee, M., Ackermans, S., van As, N., Chang, H., Lucas, E., & Ijsselsteijn, W. (2019)**.** Caring for vincent: a chatbot for self-compassion. In Brewster, S. et al. (Eds.). Proceedings of the Conference on Human Factors in Computing Systems (CHI '19), 1–13. doi:10.1145/3290605.3300932

Lee, S., Lee, N., & Sah, Y. J. (2019)**.** Perceiving a mind in a chatbot: Effect of mind perception and social cues on co-presence, closeness, and intention to use. *International Journal of Human–Computer Interaction*, *36*, 930–940. doi:10.1080/10447318.2019.1699748

Lee, Y.-C., Yamashita, N., Huang, Y., & Fu, W. (2020)**.** "I hear you, I feel you": Encouraging deep self-disclosure through a chatbot. *CHI '20: CHI Conference on Human Factors in Computing Systems.* In Bernhaupt, R. et al. (Eds.). Proceedings of the ACM CHI Conference on Human Factors in Computing Systems (CHI '20), 1–12. doi:10.1145/3313831.3376175

Lisetti, C. L., Amini, R., Yasavur, U., & Rishe, N. (2013)**.** I can help you change! An empathic virtual agent delivers behavior change health interventions. *ACM Transactions on Management Information Systems*, *4*(4), 1–28. doi:10.1145/2544103

Liu, B., & Sundar, S. S. (2018)**.** Should machines express sympathy and empathy? Experiments with a health advice chatbot. *Cyberpsychology, Behavior and Social Networking*, *21*(10), 625–636. doi:10.1089/cyber.2018.0110

Malhotra, A., Hoey, J., König, A., & Vuuren, S. (2016)**.** A study of elderly people's emotional understanding of prompts given by virtual humans. *10th EAI International Conference on Pervasive Computing Technologies for Healthcare.* In Favela, J. et al. (Eds.). Proceedings of the 10th EAI International Conference on Pervasive Computing Technologies for Healthcare, 13–16. doi:10.4108/eai.16-5-2016.2263327

McDonnell, M., & Baxter, D. (2019)**.** Chatbots and gender stereotyping. *Interacting with Computers*, *31*(2), 116–121. doi:10.1093/iwc/iwz007

Nguyen, H., & Masthoff, J. (2007)**.** Is it me or is it what I say? Source image and persuasion. In Hutchison, D. et al. (Eds.). Proceedings of the 2nd International Conference on Persuasive Technology (PERSUASIVE '07), *4744*, 231–242. doi:10.1007/978-3-540-77006-0_29

Nguyen, H., & Masthoff, J. (2009)**.** Designing empathic computers: The effect of multimodal empathic feedback using animated agent. In Chatterjee, S. et al. (Eds.). Proceedings of the 4th International Conference on Persuasive Technology (Persuasive '09), 7–16. doi:10.1145/1541948.1541958

Olafsson, S., Kimani, E., Asadi, R., & Bickmore, T. W. (2017)**.** That's a rap: Increasing engagement with rap music performance by virtual agents. In Beskow, J. et al. (Eds.). Proceedings of the 17th International Conference on Intelligent Virtual Agents (IVA '17), *Lecture Notes in Computer Science, 10498*, 325–334. doi:10.1007/978-3-319-67401-8_41

Parmar, D., Olafsson, S., Utami, D., & Bickmore, T. W. (2018)**.** Looking the part: The effect of attire and setting on perceptions of a virtual health counselor. *IVA '18: International Conference on Intelligent Virtual Agents.* In Bogdanovych, A. (Ed.). Proceedings of the 18th

International Conference on Intelligent Virtual Agents (IVA '18), 301–306. doi:10.1145/3267851.3267915

Rapp, A., Curti, L., & Boldi, A. (2021)**.** The human side of human-chatbot interaction: A systematic literature review of ten years of research on text-based chatbots. *International Journal of Human-Computer Studies*, *151*, 102630. doi:10.1016/j.ijhcs.2021.102630

Ring, L., Utami, D., & Bickmore, T. W. (2014)**.** The right agent for the job? The effects of agent visual appearance on task domain. In Bickmore, T. W. et al. (Eds.). Proceedings of the International Conference on Intelligent Virtual Agents (IVA '14), *Lecture Notes in Computer Science*, 374–384. doi:10.1007/978-3-319-09767-1_49

Robertson, S., Solomon, R., Riedl, M., Gillespie, T. W., Chociemski, T., Master, V., & Mohan, A. (2016)**.** The visual design and implementation of an embodied conversational agent in a shared decision-making context (ecoach). In Zaphiris, P. et al. (Eds.). Proceedings of the 3rd International Conference on Learning and Collaboration Technologies, *Lecture Notes in Computer Science, 9753*, 427–437. doi:10.1007/978-3-319-20609-7_40

Schmeil, A., & Suggs, S. (2014)**.** "How am I doing?" - personifying health through animated characters. In Marcus, A. (Ed.). Proceedings of the 3rd International Conference on Design, user experience, and usability (DUXU '14), *Lecture Notes in Computer Science*, 91–102. doi:10.1007/978-3-319-07635-5_10

Shi, W., Wang, X., Oh, Y. J., Zhang, J., Sahay, S., & Yu, Z. (2020)**.** Effects of persuasive dialogues: Testing bot identities and inquiry strategies. *CHI '20: CHI Conference on Human Factors in Computing Systems.* In Bernhaupt, R. et al. (Eds.). Proceedings of the ACM CHI Conference on Human Factors in Computing Systems (CHI '20), 1–13. doi:10.1145/3313831.3376843

Silverman, B. G., Holmes, J., Kimmel, S., Branas, C., Ivins, D., Weaver, R., & Chen, Y. (2001)**.** Modeling emotion and behavior in animated personas to facilitate human behavior change: The case of the heart-sense game. *Health Care Management Science*, *4*(3), 213–228. doi:10.1023/a:1011448916375

Skalski, P., & Tamborini, R. (2007)**.** The role of social presence in interactive agent-based persuasion. *Media Psychology*, *10*(3), 385–413. doi:10.1080/15213260701533102

Ter Stal, S., Kramer, L. L., Tabak, M., op den Akker, H., & Hermens, H. (2020)**.** Design features of embodied conversational agents in ehealth: A literature review. *International Journal of Human-Computer Studies*, *138*, 102409. doi:10.1016/j.ijhcs.2020.102409

Van Vugt, H. C., Konijn, E. A., Hoorn, J. F., & Veldhuis, J. (2006)**.** Why fat interface characters are better e-health advisors. In Gratch, J. (Ed.). Proceedings of the International Conference on Intelligent Virtual Agents (IVA '06), *4133*, 1–13. doi:10.1007/11821830_1

Van Wissen, A., Vinkers, C., & van Halteren, A. (2016)**.** Developing a virtual coach for chronic patients: A user study on the impact of similarity, familiarity and realism. In Meschtscherjakov, A. et al. (Eds.). Proceedings of the 11th International Conference on Persuasive Technology (PERSUASIVE '16), *Lecture Notes in Computer Science, 9638*, 263–275. doi:10.1007/978-3-319-31510-2_23

Westerman, D., Cross, A. C., & Lindmark, P. G. (2019)**.** I believe in a thing called bot: Perceptions of the humanness of "chatbots". *Communication Studies*, *70*(3), 295–312. doi:10.1080/10510974.2018.1557233

Wixom, B. H., & Todd, P. A. (2005)**.** A theoretical integration of user satisfaction and technology acceptance. *Information Systems Research : ISR*, *16*(1), 85–102. doi:10.1287/isre.1050.0042

Yin, L., Bickmore, T. W., & Cortés, D. E. (2010)**.** The impact of linguistic and cultural congruity on persuasion by conversational agents. In Allbeck, J. (Ed.). Proceedings of the 10th International Conference on Intelligent Virtual Agents (IVA '10), *Lecture Notes in Computer Science, 6356*, 343–349. doi:10.1007/978-3-642-15892-6_36

Zhou, S., Bickmore, T. W., Paasche-Orlow, M. K., & Jack, B. W. (2014)**.** Agent-user concordance and satisfaction with a virtual hospital discharge nurse. In Bickmore, T. W. et al. (Eds.). Proceedings of the 14th International Conference on Intelligent Virtual Agents (IVA '14), *Lecture Notes in Computer Science, 8637*, 528–541. doi:10.1007/978-3-319-09767-1_63

Zhou, S., Zhang, Z., & Bickmore, T. W. (2017)**.** Adapting a persuasive conversational agent for the chinese culture. *2017 International Conference on Culture and Computing (Culture and Computing).* Proceedings of the International Conference on Culture and Computing, 89–96. doi:10.1109/Culture.and.Computing.2017.42
